# Molecular model of a sensor of two-component signaling system

**DOI:** 10.1038/s41598-021-89613-6

**Published:** 2021-05-24

**Authors:** Yury L. Ryzhykau, Philipp S. Orekhov, Maksim I. Rulev, Alexey V. Vlasov, Igor A. Melnikov, Dmytro A. Volkov, Mikhail Yu. Nikolaev, Dmitrii V. Zabelskii, Tatiana N. Murugova, Vladimir V. Chupin, Andrey V. Rogachev, Andrey Yu. Gruzinov, Dmitri I. Svergun, Martha E. Brennich, Ivan Yu. Gushchin, Montserrat Soler-Lopez, Arne Bothe, Georg Büldt, Gordon Leonard, Martin Engelhard, Alexander I. Kuklin, Valentin I. Gordeliy

**Affiliations:** 1 Research Center for Molecular Mechanisms of Aging and Age-Related Diseases, Moscow Institute of Physics and Technology, 141700 Dolgoprudny, Russia; 2 Structural Biology Group, European Synchrotron Radiation Facility, 71 Avenue des Martyrs, 38000 Grenoble, France; 3 Institute of Biological Information Processing (IBI-7: Structural Biochemistry), Forschungszentrum Jülich, 52425 Jülich, Germany; 4 JuStruct: Jülich Center for Structural Biology, Forschungszentrum Jülich, 52428 Jülich, Germany; 5 Institute of Crystallography, University of Aachen (RWTH), Jaegerstrasse 17-19, 52056 Aachen, Germany; 6 Frank Laboratory of Neutron Physics, Joint Institute for Nuclear Research, 141980 Dubna, Russia; 7 European Molecular Biology Laboratory, Hamburg Outstation, 22607 Hamburg, Germany; 8 Synchrotron Crystallography Team, EMBL Grenoble Outstation, 71 Avenue des Martyrs, 38042 Grenoble, France; 9 Department Structural Biochemistry, Max Planck Institute of Molecular Physiology, 44227 Dortmund, Germany; 10 Institut de Biologie Structurale Jean-Pierre Ebel, Université Grenoble Alpes-Commissariat à l’Energie Atomique et aux Energies Alternatives-CNRS, 38027 Grenoble, France

**Keywords:** Membrane proteins, SAXS, Computer modelling, Cell signalling

## Abstract

Two-component systems (TCS) are widespread signaling systems present in all domains of life. TCS typically consist of a signal receptor/transducer and a response regulator. The receptors (histidine kinases, chemoreceptors and photoreceptors) are often embedded in the membrane and have a similar modular structure. Chemoreceptors were shown to function in highly ordered arrays, with trimers of dimers being the smallest functional unit. However, much less is known about photoreceptors. Here, we use small-angle scattering (SAS) to show that detergent-solubilized sensory rhodopsin II in complex with its cognate transducer forms dimers at low salt concentration, which associate into trimers of dimers at higher buffer molarities. We then fit an atomistic model of the whole complex into the SAS data. The obtained results suggest that the trimer of dimers is "tripod"-shaped and that the contacts between the dimers occur only through their cytoplasmic regions, whereas the transmembrane regions remain unconnected.

## Introduction

**Figure 1:**
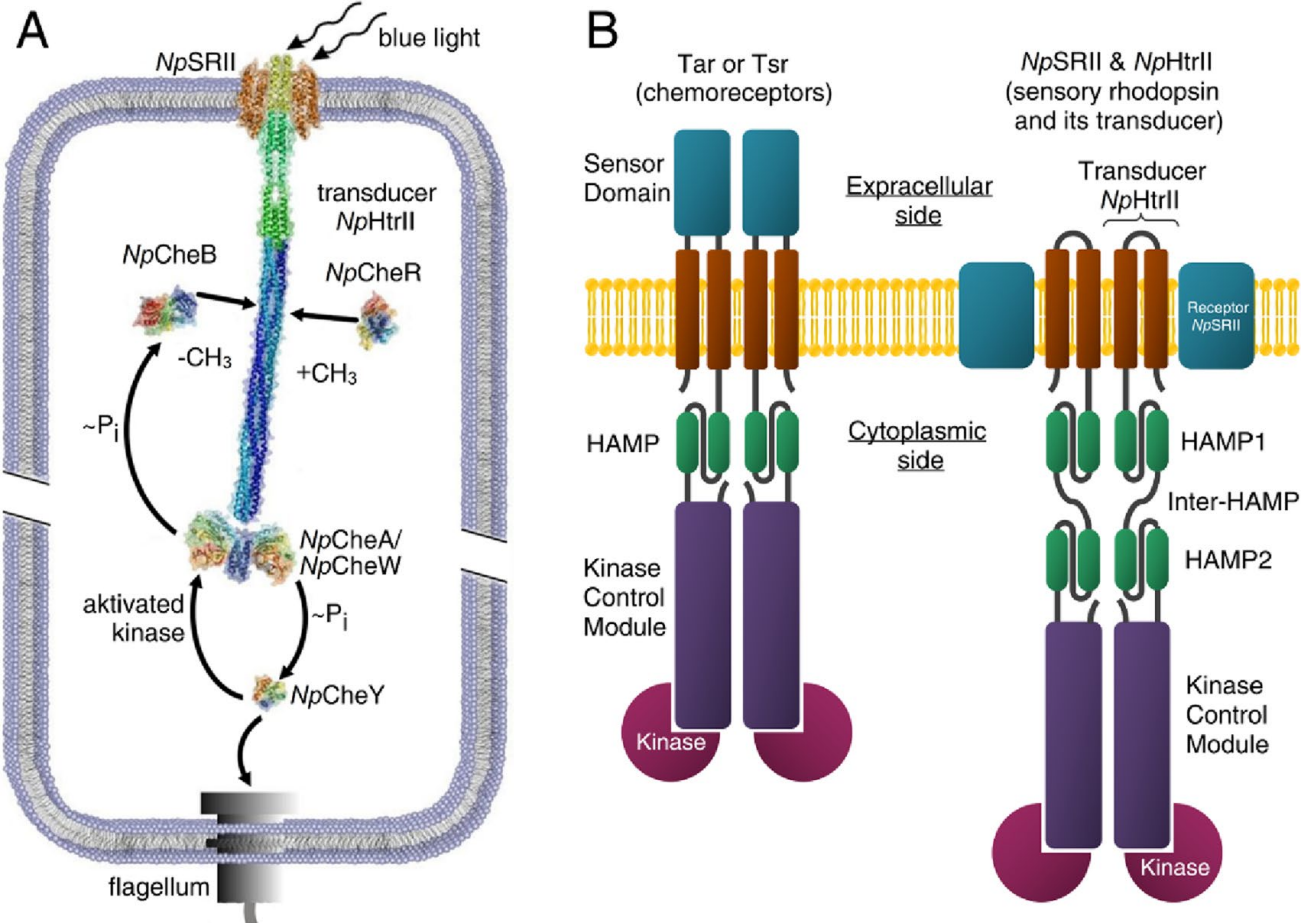
Signal transduction pathway in case of the two-component phototaxis system of *Natronomonas pharaonis*^5^ and domain architecture of membrane chemo- and photoreceptors of TCS. (**A**) Light activated sensory rhodopsin II (*Np*SRII) induces conformational and/or dynamical changes in the transducer (*Np*HtrII), which are converted by two HAMP domains and conveyed along the 200 Å long transducer to the tip region. Activated by the transducer histidine kinase CheA (bound to the adapter protein CheW) undergoes auto-phosphorylation and further transfers the phosphate group to the response regulators CheY or CheB. CheY affects the rotational bias of the flagellar motor, while the methylesterase CheB along with the methyltransferase CheR controls the adaptation mechanism. (**B**) Cartoon representations of the chemoreceptor dimer (Tar and Tsr in complex with kinases) from *E. coli* and of the photosensor dimer of the complex of the sensory rhodopsin II with its cognate transducer *Np*HtrII and kinases from *N. pharaonis*.

Two-component systems (TCS) are present in all domains of Life. They are the most common signaling systems in prokaryotes but are absent in mammals, making them potential antimicrobial drug targets^1^. A recent work indicated that TCS networks expressed in mammalian cells might pave the way for orthogonal signaling^2^. However, this will require more atomic level detail concerning their structure/function relationships. In this regard, detailed information has been obtained for chemotaxis and photoreceptor TCS of motile enteric bacteria and archaea, respectively^3,4^. This family of TCS consist of a transmembrane receptor, which interacts with a His-kinase (CheA) and adaptor proteins (CheW) attached at their cytoplasmic tips. The response regulators CheY and CheB are phosphorylated by CheA and function either as a switch factor for the flagellar motor (CheY) or as an adaptation component (CheB)^5^ (Fig. 1A). Chemotaxis receptors of motile bacteria form homodimers comprised of extracellular sensor domain and transmembrane regions, and a long rod-shaped cytoplasmic domain. For photoreceptors, the sensor domain constitutes a microbial rhodopsin which forms a 2:2 complex with its cognate transducer (Htr). Transducers have a long cytoplasmic domain, similarly to chemoreceptors, with which they share a high level of homology^4,6^ (Fig. 1B). In literature, the term "sensor" usually means the first component of the two-component system. In the case of chemotaxis, it is a chemoreceptor, which has the so-called sensory domain. In the case of a photoreceptor, the sensory domain is represented by a sensory rhodopsin. Thus, the complex of a sensory rhodopsin with the transducer plays the role of a sensor of TCS. We use the term "sensor" in our work meaning that it is the first part of the phototaxis TCS.

The cytoplasmic domain of Htr has a modular design comprising a transmembrane region, two HAMP domains (connected by a helical inter-HAMP region^7^) and an extended adaptation module bearing reversible methylation sites and containing a Gly-hinge. The adjacent signaling region harbors the binding sites for the adaptor protein CheW and the histidine kinase CheA. Interestingly, chemoreceptors from enteric bacteria possess only one HAMP-domain^8^ in contrast to archaeal transducers^9^.

Despite a wealth of biochemical data, the molecular mechanism of the signal transduction by TCSs is still to be fully revealed. A common understanding concerns the alternating dynamics of the cytoplasmic domains. Both chemoreceptors and Htrs display different dynamics in adjoining modules, which has been correlated with the signal transfer along the cytoplasmic rod^5,10^. A possible role of hinges between the modules could be the steric accommodation of the dimers into trimers of dimers and oligomers of higher order^11,12^. These trimers of dimers constitute the functional unit capable of activating CheA^13^. Together with CheA and CheW, trimers form the core unit of the extended signaling arrays^14^, compact membrane-attached assemblages of signaling units responsible for amplification of the incoming stimulus^15–17^.

An important *sine qua non* of a full understanding the mechanism of signal transfer along the trimers of dimers is a high-resolution structural information for a full-length TCS sensor. However, three-dimensional, near atomic-resolution X-ray structures have only been published for fragments of histidine kinases^9,18–20^ and chemoreceptors^21–23^, and for a truncated phototaxis-transducer complex from *N. pharaonis*^24^. For this latter, NMR data are also available^25^. Also, several atomistic models of *E. coli* chemoreceptors were built based on low-resolution EM data, particularly, the homodimer of the aspartate receptor Tar^11^, the trimers-of-dimers of the cytoplasmic domains of serine chemoreceptors in different signaling states^26^, and the chemosensory array core signaling unit formed by a mixture of receptors with different adaptational modifications^27^. This insufficiency of high-resolution descriptions of the structures of full-length TCS sensors is probably due to the inherent flexibility of their cytoplasmic domains^5,10,11^. However, small-angle scattering (SAS) is a low-resolution method, which can be highly efficient when combined with available high-resolution structures of protein fragments using computer modeling^28,29^. An important advantage of SAS is that structural data can be collected for solubilized protein complexes in conditions close to their native environment.

*Np*SRII/*Np*HtrII mediates negative phototaxis in halobacterial *N. pharaonis* and represents a model TCS sensor and, similarly to chemoreceptors^30^, the *Np*SRII/*Np*HtrII complex forms trimers of dimers in the *N. pharaonis* membrane^31^. *N. pharaonis* grows optimally at 3.5 M NaCl^32^. It has been shown that structure and oligomerization state of the *Np*SRII/*Np*HtrII strongly depend on salt concentration^33^. Here, we describe the full-length structure of a sensory rhodopsin II/transducer complex (*Np*SRII/*Np*HtrII) by integrating published high-resolution structural data with SAS measurements. Structural information was gained for *Np*SRII in complex with the transmembrane domain of *Np*HtrII, which show a dimeric organization in different activated states^24,34,35^. For the rest of the *Np*HtrII, homologues were chosen including the HAMP domains of Aer2 cytoplasmic sensor^23^ and for the hypothetical transmembrane receptor AF1503^36^, sensor histidine kinase NarQ^18–20,37^, kinase control modules including the methyl-accepting chemotaxis protein I (Tsr) from *E. coli*^22^ and the chemotaxis protein from *Thermatoga maritima*^38,39^. Our work both confirms the dependence of oligomeric state on ionic strength and provides molecular models of the dimeric and "trimer of dimers" forms of the full-length complex. In our manuscript, we used the term "molecular model" meaning a model, which is constructed by molecular modeling with the high-resolution structures of the fragments of full-length protein (transmembrane domain, HAMP1 and HAMP2 domains and Kinase control module) under low resolution SAS constraints and verified with available biochemical and biophysical literature data. The results obtained allow us to suggest a "tripod"-shaped model for the full-length *Np*SRII/*Np*HtrII trimer of dimers in which the dimers associate solely through contacts between their cytoplasmic domains.

## Ionic strength dependence of the oligomerization state of the *Np*SRII/*Np*HtrII complex

In order to investigate conditions, under which trimers of dimers are formed, we performed SAXS (small angle X-ray scattering), SANS (small angle neutron scattering) and CD (circular dichroism) experiments (Fig. S1) at different salt concentrations on solutions of detergent solubilized [n-dodecyl-β-d-maltopyranoside (DDM)] full-length *Np*SRII/*Np*HtrII as well as with truncated *Np*SRII/*Np*HtrII_137_. *Np*HtrII_137_ comprises the transmembrane region (1–83 a. a.) and the HAMP1 domain (84–136 a. a.) of *Np*HtrII.

**Figure 2:**
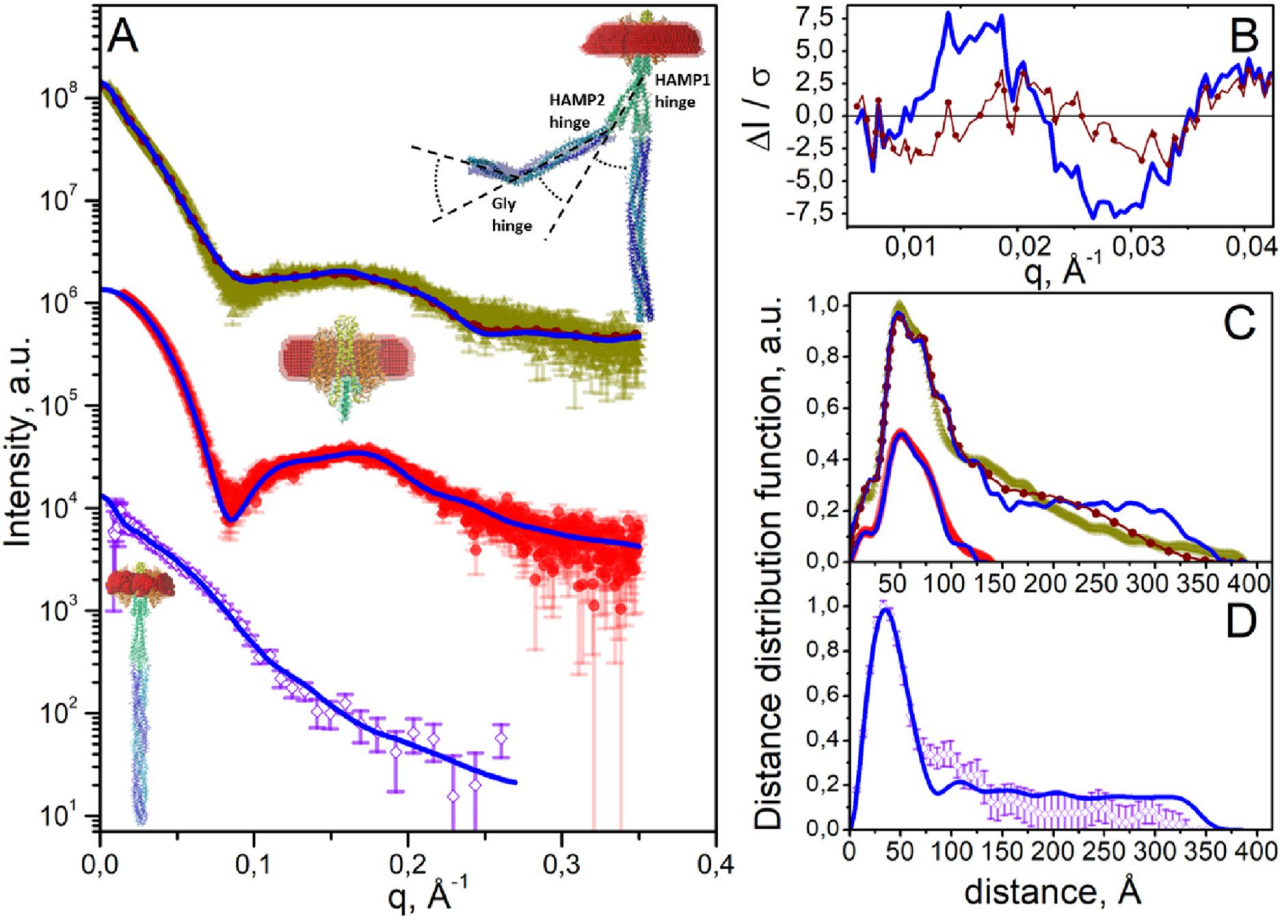
Dimerization of *Np*SRII/*Np*HtrII_137_ and of full-length *Np*SRII/*Np*HtrII at low salt concentration. (**A**) Bottom—experimental SANS curve for *Np*SRII/*Np*HtrII (purple hollow rhombus) and CRYSON fit (χ^2^ = 0.7, corresponding blue line) based on a model (shown on the left of the curves) of an *Np*SRII/*Np*HtrII dimer with a detergent corona. Middle—experimental SAXS curve for the *Np*SRII/*Np*HtrII_137_ dimer (red circles) and MEMPROT fit (χ^2^ = 1.5, corresponding blue line) based on a model (shown near the corresponding curve) of the *Np*SRII/*Np*HtrII_137_ dimer with the detergent corona. Top—experimental SAXS curve for full-length *Np*SRII/*Np*HtrII (dark yellow triangles) and two theoretical approximations. The first (blue solid line) is a CRYSOL3 fit (χ^2^ = 5.1) based on a "straight" model of the *Np*SRII/*Np*HtrII dimer. The second (brown pointed line) is a fit (χ^2^ = 3.4) based on a combination of modified models of the *Np*SRII/*Np*HtrII dimer (see “Methods”, Fig. S2 and Text document S1 for details). While the difference between the two fits is not immediately apparent, the improved fit of the second approximation is evident when considering of the relative residuals of the fit in the region in reciprocal space q < 0.04 Å^−1^ (see **B**), that corresponds to the distances > 160 Å in a real space. For this range of distances, discrepancy between the experimental distance distribution function and theoretical one obtained for the "straight" model of the *Np*SRII/*Np*HtrII dimer is evident (see **C**). In the representations of the atomic models, the detergent belt is shown in red. (**B**) Relative residuals of theoretical approximations and experimental SAXS data obtained for full-length *Np*SRII/*Np*HtrII (**A**, top). Data related to the "straight" model is shown as blue solid line; data related to the combination of modified models is shown as brown pointed line. (**C**) Distance distribution functions calculated from the SAXS curves shown in (**A**) (designations are the same as in **A**). (**D**) Distance distribution functions calculated from SANS data shown in (**A**) (designations are the same as in **A**).

At low salt conditions SAXS scattering curves for the truncated complex were fitted well (χ^2^ = 1.5) using a model of a dimer of *Np*SRII/*Np*HtrII_137_ surrounded by a detergent belt (see “Methods” for the details) (Fig. 2A, middle, Table S1), strongly suggesting that, under the conditions employed, interactions between the membrane and/or HAMP1 domains of *Np*HtrII_137_ are sufficient to induce dimerization.

A fit based on an optimized dimer model (see “Methods” for the modeling details) containing a "straight" adaptational domain (Fig. 2A, bottom) showed reasonable (χ^2^ = 0.7) agreement with experimental low salt condition SANS data (this also applies to the matching of the distance distribution functions, see Fig. 2D). Hovewer, complementary SAXS experiments, performed as for *Np*SRII/*Np*HtrII_137_ at a synchrotron source, suggested that the optimized model featuring the "straight" adaptation domain does not fully describe the overall structure of a full-length *Np*SRII/*Np*HtrII dimer. Here, a fit between the experimental scattering curve and that calculated from the "straight transducer" model yields χ^2^ = 5.1. This can be improved (χ^2^ = 3.4) using a calculated scattering curve obtained by combining a series of models in which bends of between − 90° and 90° (with the step of 30°) were induced in the HAMP1-, HAMP2- and Gly-regions (see “Methods”, Fig. 2, Fig. S2 and Supplementary Text document S1 for details). This procedure produced a noticeably improved fit of the distance distribution functions calculated from the experimental and theoretical scattering curves, particularly in the 160–370 Å region (Fig. 2C). This range of distances corresponds in reciprocal space to the range of q < 0.04 Å^−1^, in which there is a significant improvement in agreement with experimental SAXS data when the flexible hinges model is used (see Fig. 2B). This observation suggests presence of multiple conformational states of full-length *Np*SRII/*Np*HtrII dimers that can be described as structural polydispersity of the model that is in line with EM studies^11^ showing that bending of Tar chemoreceptor dimers occurs at flexible HAMP and/or Gly hinges. Moreover, it has also been suggested that this bending may play a crucial role in the formation of the hexagonal arrays of membrane receptors in complex with kinases CheA and CheW^12^. Nevertheless, we cannot rule out that such conformational flexibility may be a result of low ionic strength in which the transducer domain is partially disordered. Indeed, in line with the results of other studies^33,40^, CD-spectra of the full-length *Np*SRII/*Np*HtrII complex under different salt conditions (Fig. S1) suggest partial disordering of *Np*SRII/*Np*HtrII dimers.

**Figure 3:**
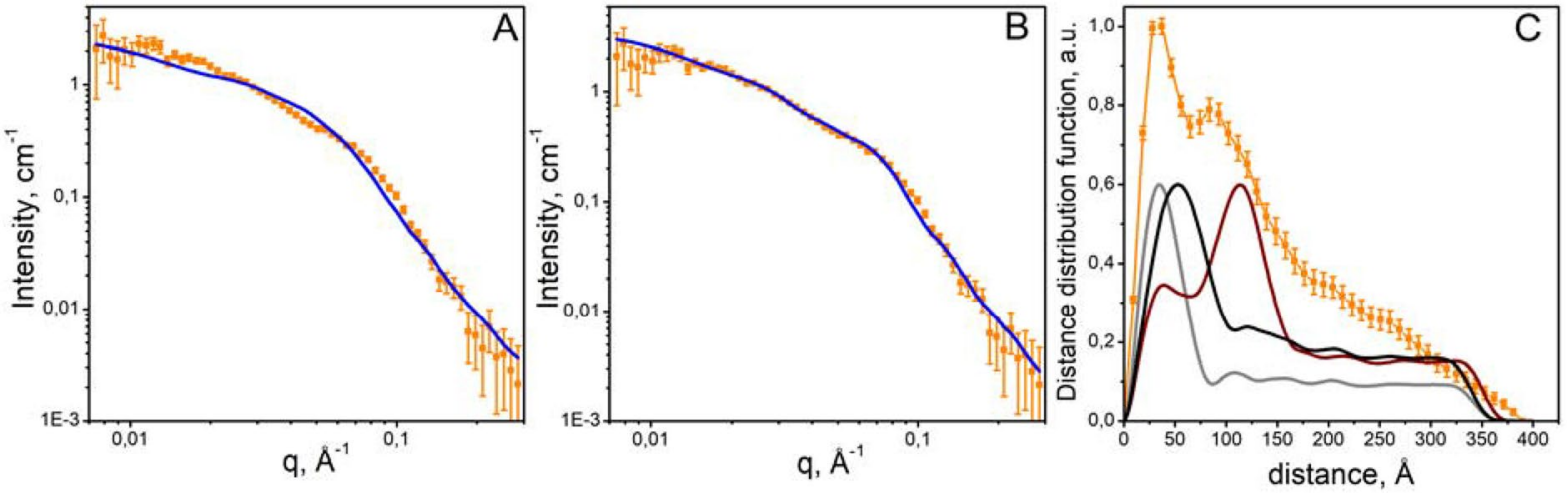
SANS curve for *Np*SRII/*Np*HtrII at 4.0 M NaCl and corresponding distance distribution functions. (**A**) Experimental scattering curve for *Np*SRII/*Np*HtrII at 4.0 M NaCl (orange squares) fitted with χ^2^ = 5.5 to a theoretical curve (blue line) calculated for a mixture of *Np*SRII/*Np*HtrII dimers and trimers of dimers which inter-dimer contacts are induced both between the transmembrane regions of dimers and their cytoplasmic tips (Fig. 4A). (**B**) Experimental scattering curve for *Np*SRII/*Np*HtrII at 4.0 M NaCl (orange squares) fitted with χ^2^ = 1.3 to a theoretical curve (blue line) calculated for a mixture of *Np*SRII/*Np*HtrII dimers and "tripod"-shaped trimers of dimers (Fig. 4B). (**C**) Distance distribution function calculated from the experimental curve shown in (**A**,**B**) (orange squares), and theoretical distance distribution functions of the dimers (grey line), the "tripod"-shaped (Fig. 4B) trimers of dimers (brown line) and the "transmembrane-bound" conformation of trimer of dimers of the *Np*SRII/*Np*HtrII (Fig. 4A) (black line). For greater clarity, distance distribution functions were normalized to obtain maximum values of 1.0 for the experimental and 0.6 for the theoretical curves.

**Figure 4:**
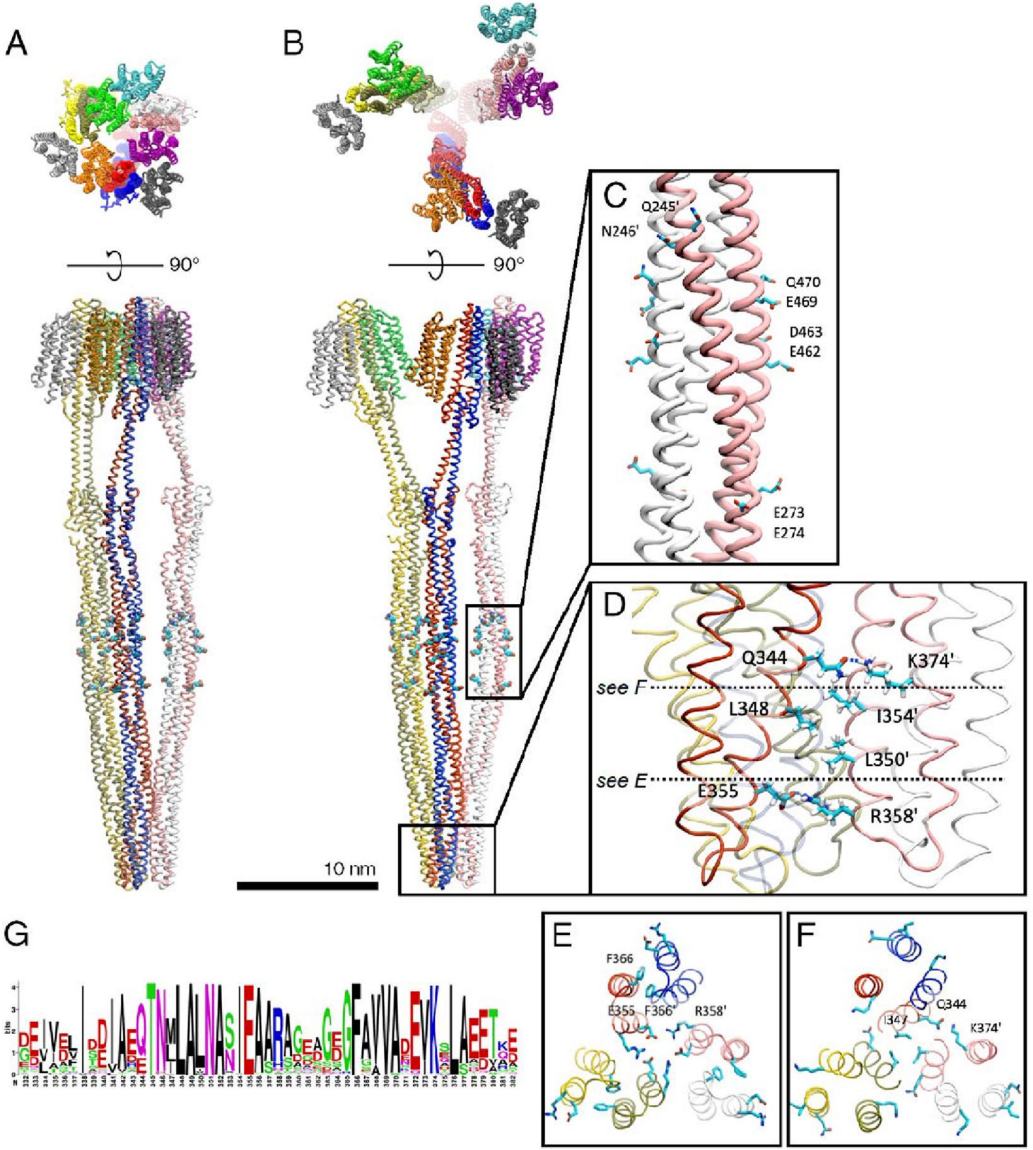
Optimized molecular models of the *Np*SRII/*Np*HtrII trimer of dimers. (**A,B**) Molecular models of the "transmembrane-bound" (**A**) and the "tripod"-shaped (**B**) trimer of dimers. Individual polypeptide chains are colored differently. Putative methylation sites are represented as spheres. (**C**) Putative methylation sites in the methyl-accepting region of a single dimer of the complex. (**D**) Inter-dimer contacts within the highly conservative tip region of the cytoplasmic domain of the "tripod"-shaped trimer of dimers. Key amino acid residues are shown with those belonging to the partnering dimer labeled by apostrophe. (**E**) Cross-section view highlighting the formation of ionic locks between E355-R358' (homologous to E385 and R388' from Tsr^42^ and stacking between F366 (equivalent to F396 of Tsr) and F366'. (**F**) Cross section depicting contacts between conservative K374' and Q344 residues, and hydrophobic contacts between I347. (**G**) Logo plot showing conservation of residues in the tip region involved in the formation of the trimer contacts.

While both SAXS and SANS experiments clearly suggest that *Np*SRII/*Np*HtrII mainly forms dimers at low ionic strength, this is not the case at increasing salt concentrations. Figure 3A shows the SANS scattering curve for the full-length *Np*SRII/*Np*HtrII measured in a buffer containing 4.0 M NaCl. This cannot be satisfactorily matched by a theoretical curve calculated from the polydisperse dimers described above and to obtain a reasonable fit it was necessary to calculate a theoretical scattering curve based upon a mixture *Np*SRII/*Np*HtrII dimers and trimers of dimers. Two different forms of trimers of dimers were constructed for use in our calculations. Firstly, similar to a previously proposed model of the trimer of dimers^5^, the direction of bending angles at the HAMP and Gly hinges was such that inter-dimer contacts are induced both between the transmembrane regions of dimers and their cytoplasmic tips (see Fig. 4A, Fig. S3). For the second model, the direction of bending angles at the HAMP and Gly hinges was introduced such that the transmembrane regions at the base of the trimer of dimers adopt a tripod-type disposition and inter-dimer contacts are formed only between the tips of the cytoplasmic domains of each dimer (Fig. 4B). In both cases, the detergent belt was applied as described for the SANS analysis at the low salt (see above, Fig. 2A, bottom). Fitting the experimental SANS curve obtained for *Np*SRII/*Np*HtrII at 4.0 M NaCl to a theoretical curve based on a mixture of *Np*SRII/*Np*HtrII dimers and trimers of dimers, inter-dimer contacts in which are induced both between the transmembrane regions of dimers and their cytoplasmic tips (Fig. 4A), resulted in χ^2^ = 5.4 (Fig. 3A). In contrast, fitting the same SANS curve to a theoretical curve based on a mixture of *Np*SRII/*Np*HtrII dimers and "tripod"-shaped trimers of dimers (Fig. 4B) resulted in χ^2^ = 1.3 (Fig. 3B). Pair distance distribution function calculated from experimental SANS data obtained at 4.0 M NaCl has two peaks (see Fig. 3C). The position of the first peak (r ~ 30 Å) allows one to interpret it as a peak from dimers, while the position of the second peak (r ~ 100 Å) is in a good agreement with the position of the peak corresponding to the "tripod"-shaped model of the trimer of dimers (see Fig. 3C). The conformation of trimer of dimers of the *Np*SRII/*Np*HtrII SANS experiments at NaCl concentrations 1.4 M and 2.8 M (Fig. S4) produced similar results but indicated an increasing fraction of tripod-style trimers of dimers with increasing salt concentration. Resulting weight fractions of "tripod"-shaped trimers of dimers are 18 ± 3%, 28 ± 2% and 36 ± 2% for 1.4 M, 2.8 M and 4.0 M, respectively (Fig. S5, Table S2). These results support that the formation of *Np*SRII/*Np*HtrII trimers of dimers at high salt concentration is likely to occur through the contacts of the cytoplasmic tips of dimers with their transmembrane parts remaining unconnected (Fig. 4B).

## A molecular model of the full-length *Np*SRII/*Np*HtrII complex

The tripod-like model of the structure of the *Np*SRII/*Np*HtrII trimer of dimers (Fig. 4B) which provides the best fit to the experimental SANS scattering curve obtained at 4.0 M NaCl (Fig. 3B) was constructed based on the high-resolution structures of individual fragments of the TCSs sensors from *E. coli*, *N. pharaonis*, and *T. maritima*^18,22,24,34,35,37–39^. The initial model obtained was further optimized using molecular dynamics simulations (see “Methods”). In the resulting model, the transmembrane domains of individual dimers are separated but their cytoplasmic tips form a tight interface. As expected, given that modelling of the cytoplasmic domain was based on the X-ray structure of the highly conserved interface of *E.coli* Tsr^22^, dimer-dimer interactions (Fig. 4D) are mediated by a number of inter-residue contacts involving amino acids conserved across homologs of *Np*HtrII (see logo plot in Fig. 4G). For example, E355 of one dimer forms a salt bridge with R358' of a neighboring dimer (Fig. 4E); a compact hydrophobic patch is formed by L348, I354' and L350', hydrogen bonds are formed between Q344 and K374' (Fig. 4F). In addition, stacking between the aromatic rings of F366-F366' stabilizes the intra-dimer interface similar to bacterial chemoreceptors^41^ (Fig. 4E).

## Discussion

In present study, the combination of SAS and molecular modeling has allowed us to propose a molecular model of the full-length phototaxis complex from the halophilic archaeon *N. pharaonis* and to reveal the effect of salt concentration on its oligomeric state and dynamics.

We verified our molecular model with available biochemical and biophysical literature data. The monomer–monomer interactions between transducers are in accordance with the following high-resolution structural data. The dimer of the transmembrane domain is based on its available structure (PDB code 1h2s^24^). Dimers of HAMP1 and HAMP2 domains are constructed according to homology with HAMP domain of NarQ from *E.coli* (PDB code 5jeq^18^). The cytoplasmic domain (kinase control module) is presented by the coiled-coil of two antiparallel helices connected by a "U-turn", as it was shown for cytoplasmic domains of TsrQ from *E.coli* (PDB code 1qu7^22^), MCP from *Termatoga maritima* (PDB code 2ch7^38^), etc. In these cases, coiled-coil structure stabilized by interactions between the extended interfaces consisting of hydrophobic amino acids, mainly leucines. Dimer–dimer interactions (Fig. 4D) are mediated by a number of inter-residue contacts involving amino acids conserved across homologs of *Np*HtrII (see logo plot, Fig. 4G).

Positions of highly conservative residues 345–355 in the cytoplasmic tip domain of *Np*HtrII in our molecular model of the *Np*SRII/*Np*HtrII trimer of dimers are in a good agreement with the EPR studies by Orban-Glaß et al.^43^. In that work, the dynamic and structural properties of the cytoplasmic tip domain of *Np*HtrII were investigated using site-directed spin labeling electron paramagnetic resonance spectroscopy. Distance distributions obtained by double electron–electron resonance typically have three peaks, which can be interpreted as three characteristic inter-spin distances that correspond to three groups of inter-residue distances (see Fig. S6A,B). This finding supports the fact that archaeal photoreceptor/transducer complexes form trimers of dimers analogous to methyl-accepting chemotaxis proteins at high salt concentrations. Comparison of the distance between the residues labeled with a spin label showed that the distance between the nearest isoleucines I347 decreases with an increase in the salt concentration from 500 mM to 2 M NaCl (Fig. S6C). This rearrangement indicates that a trimer of dimers is formed with I347 facing inside the resulting structure (see Fig. 4F, Fig. S6A), playing an important role in the formation of inter-dimer contact due to hydrophobic interaction.

At low ionic strength (150 mM NaCl), our SAS data are most compatible with a dimeric conformation for both the truncated *Np*SRII/*Np*HtrII_137_ and for full-length *Np*SRII/*Np*HtrII. In the latter, the very long cytoplasmic domain of the dimeric *Np*HtrII transducer appear to be highly flexible, corroborating the intrinsically dynamic nature of chemo- and photoreceptors described in some recent publications (e.g.^10,40,44^). Coupled with the conclusions of a previous study that the cytoplasmic domain of *Np*HtrII's (*p*HtrII-cyt) does not form dimers at low ionic strength^33^, our results imply that only the transmembrane region is required for dimerization of *Np*SRII/*Np*HtrII. However, this observation is in contrast to that obtained for a similar construct (*Np*SRII/*Np*HtrII_157_ containing HAMP1 and inter-HAMP (137–156 a. a.) domains). This might be due to a lower detergent concentration used in the current experiments.

While the minimum construct required for *Np*SRII/*Np*HtrII to form dimers may still be a matter for debate, our SAS data obtained at low ionic strength clearly suggest that full-length *Np*SRII/*Np*HtrII forms dimers under such conditions (see Fig. 2A). In order to improve the agreement between theoretical and experimental scattering curves data, we generated models of different possible conformations of the *Np*SRII/*Np*HtrII dimers which, while maintaining an elongated shape for the *Np*HtrII dimers, considered both bending at its flexible hinges^11^ and partial unfolding^33,40^ of this domain at low salt conditions. Our results confirm a highly dynamic nature of the transducer dimer at low salt: the root mean square deviations of bending angles from the zero value are in the range from 50° to 60° for all the three HAMP1-, HAMP2-, and Gly- hinges (see Fig. S2).

For the cytoplasmic domain of the *Np*HtrII (234–504 a. a.), Budyak et al.^33^ also observed a partial random coil configuration at low salt concentrations. Due to the difficulty to unequivocally determine the amount of random coil structures, these authors argued in light of their SANS in favor a highly dynamic helical rod at low salt conditions. This finding has been confirmed by subsequent results published in the work^40^, according to which *p*HtrII-cyt has a strong propensity for helix. Firstly, *p*HtrII-cyt has a high helix propensity inferred from the sequence; *p*HtrII-cyt is predicted to be a helical coiled-coil based on a homology to the Tst-cyt^22^. Secondly, despite that at low ionic strength deconvolution of CD-spectra the secondary structure values are 77% unstructured, 20% turns and β-sheets, and only 3% α-helices, it was also shown by control experiments with helix-inducing solvent 2,2,2-trifluoroethanol (TFE) that a gradual rise in negative ellipticity at 222 nm occurs with increasing amounts of TFE. Thus, despite "random-coil" spectroscopic features, *p*HtrII-cyt can exist as a highly flexible, loosely packed but folded helical coiled-coil. The results of the comparison of CD spectra from the work^40^ with and without TFE suggest that, despite the presence of β-sheets in the deconvolution of CD spectra (see Fig. S1B), β-sheets do not necessarily have to be present in the final molecular model. Moreover, numerous algorithms for the estimation of the secondary structure composition from the CD spectra often fail to provide acceptable results on α/β-mixed or β-structure-rich proteins due to spectral variety and lower spectral amplitudes of the β-structures^45,46^. For these reasons, the secondary structure of the molecular model proposed in our work is predominantly α-helical.

Despite the fact that isolated *p*HtrII-cyt is in monomeric form under conditions of low salt concentration^33^, this domain forms dimers under these conditions when the *Np*SRII/*Np*HtrII complex is full length. The coiled-coil does not disappear, which is confirmed by the results of work^43^, where the distances between residues for the cytoplasmic tip of the full-length *Np*SRII/*Np*HtrII studied by the EPR method at low salt concentration are the same as they should be in a dimer (see Fig. S6C). These are the reasons why we used the model where the protein fragments between the flexible hinges present elongated (not globular) structures. Additionally far-UV CD data (Fig. S1) obtained in our work under low salt conditions showed conformational disordering of *Np*SRII/*Np*HtrII. The Kratky plots (Fig. S7) also confirm this: the obtained curves have maximum values at q R_G_ ~ 4 to 7, which are higher than expected for globular particles and are typical for elongated and/or flexible proteins^47,48^. After the maximum, the curves show a tendency to decrease to zero, which excludes complete unfolding of proteins^49^.

It has been shown that the chimeric proteins *Np*SRII-*Np*HtrII-Tar and *Np*SRII-*Np*HtrII-Tsr, which contain the transmembrane region and a truncated HAMP1 domain (a. a. 1–125) of the *Np*SRII/*Np*HtrII complex and the cytoplasmic domain of either *St*Tar or *Ec*Tsr, can mediate phototaxis in *E. coli*^50^. This suggests that both chimeric complexes are able to transduce signal at low salt conditions. This, taken together with our results, implies that destabilization of the remaining fragments of the cytoplasmic domains of which are not included in these chimera is the main reason for blocking the formation of the trimers of dimers of native *Np*SRII/*Np*HtrII at low ionic strength. It also suggests that at high salt concentration corresponding to the physiological range of halophilic archaea, a reordering of the cytoplasmic domains takes place and this allows the dimers to assemble into trimers of dimers, implying that the transmembrane region of *Np*SRII/*Np*HtrII complex either is not sufficient to mediate formation of trimers-of-dimers or, consistent with our "tripod"-shaped model, is not involved into in the trimerization of dimers.

The observed salt concentration-induced structural changes of the *Np*SRII/*Np*HtrII system may have two possible biological 'roles'. As pointed above, haloarchaeon *N. pharaonis* lives in the highly saline environment and the complex must be optimized to these conditions. If the phototaxis system would remain functional under the low salt conditions, it could drive the microorganisms to regions with optimal insolation regardless of the salt concentration that would ultimately lead to their death. On the other hand, we cannot also completely exclude that disordering (i.e. decreasing of secondary structure elements and increasing of conformational flexibility) of the complex may generate signal allowing the archaea to move towards higher salt concentration and avoid environments with low salinity. The salt-driven equilibrium between dynamic and compact conformations, which was observed for the *Np*HtrII HAMP1 domain by EPR^44^, supports this idea.

SANS scattering curves at higher salt concentrations (Fig. 3) clearly indicate the formation of a trimer of dimers, the fraction of which increases as does the ionic strength. Moreover, the best fit to the SANS data at higher ionic strength (Fig. 3B) is obtained by modelling a tripod-like shape, in which only the cytoplasmic tips of *Np*HtrII dimers are involved in inter-dimer contacts (Fig. 4B). This is in agreement with previous EPR studies suggesting that *Np*SRII/*Np*HtrII dimers may form oligomers of higher order (e.g., trimers of dimers) due to interactions between the transducer tips alone^43^. In our model, inter-dimer separations in the regions containing putative sites (Fig. 4C) of methylation/demethylation (determined by homology with *Hs*HtrII of *Halobacterium salinarum*^7^, see alignment in Text document S2) vary between 30 and 40 Å (Fig. S8). Thus, they are all potentially accessible to modifications by the chemotaxis methyltransferase CheR and methylesterase CheB (the radii of gyration calculated for CheR and CheB from *Salmonella typhimurium* (PDB IDs 1AF7 and 1A2O) being approximately 21 Å and 20 Å, respectively).

To the date, there is very little structural information available for full-length *E. coli* Tar/Tsr chemoreceptors or other chemoreceptors in either their demethylated (OFF) or methylated (ON) states. Our experiments provide a complete description of the demethylated complex (OFF) while a recent study by Burt et al.^27^ describes a model based on a mixture of receptors with a wide range of adaptational modifications. In the later study, the authors were able to obtain the 3D cryo-ET map of the full-length *E. coli* chemoreceptor array together with the CheW/CheA baseplate in micelles with the local resolution varying between ~ 15 and 30 Å. They further constructed all-atom models of the *E. coli* CheA.P3.P4.P5 dimer, CheW monomer, and membrane-bound, full-length Tsr homodimer using coordinates from existing high-resolution crystallographic structures where available. The two models, share remarkable similarity in the tip region, where a number of key interactions appear concurrent despite the fact that they apparently correspond to different signaling states. This is very likely due to inherent bias of the both models towards the 1QU7 structure^22^, which was obtained for the QQQQ Tsr analogue (and thus it likely corresponds to the ON-state) which was used to model the trimeric contacts of the cytoplasmic tips of dimers in both studies. On the other hand, it stems from the high structural conservation of cytoplasmic receptor arrays between Bacteria and Archaea^51^. However, the lower local resolution of the periplasmic and transmembrane regions of the model report in^27^ (apparently due to the large separation between the well-resolved cytoplasmic regions of the Tsr/Tar array and its periplasmic ligand-binding domains, combined with the relative flexibility of the cytoplasmic methylation helix bundle of the receptors) and the lack of homology between these fragments of bacterial chemoreceptors and the *Np*SRII/*Np*HtrII restrains further comparison of our results with the model of Burt et al.

**Figure 5:**
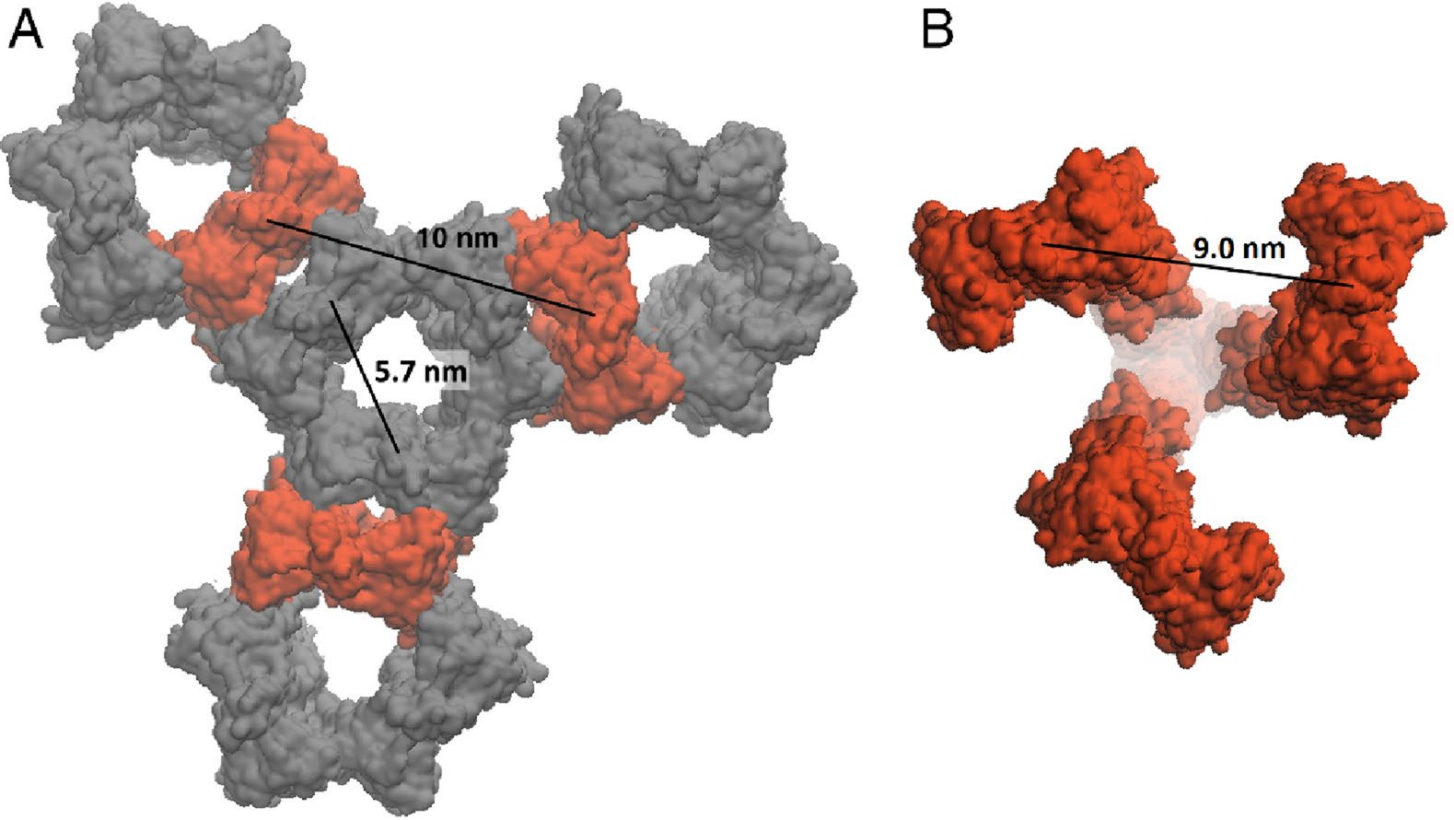
Inter-dimer distances between transmembrane parts of the *Np*SRII/*Np*HtrII dimers. (**A**) Characteristic distances between dimers in 2D-array proposed in^31^. (**B**) The "tripod"-shaped model of the trimer of dimers (Fig. 4B) demonstrating the inter-dimer distance of 9.0 nm.

Trimers of dimers are essential for the formation of large membrane signaling arrays of both photo- and chemoreceptors^14^. The absence of CheA/CheW in our experiments does not allow us to identify oligomers larger than trimers of dimers; however, our structure provides potential routes for the formation of the membrane arrays from the "tripod"-shaped trimers of dimers. One of the possibilities is that the transmembrane sensory domains of dimers within each trimer of dimers are able to form a trimeric contact in a native environment and are further packed with the neighboring trimers of dimers^31^. On the other hand, the "tripod"-shaped model for the trimer of dimers is also compatible with lattice models suggesting that trimeric contacts at the cytoplasmic part do not match trimeric contacts between the transmembrane domains (Fig. 5). Both scenarios seem feasible, taking into account the plasticity of dimers at the three flexible hinges discussed above, though the *summae* of evidences existing to the date supports the latter one. Further investigations should shed light on structural organization of photoreceptor arrays.

Here a combination of SAXS and SANS allowed us to study conditions of the trimer-of-dimers formation from the individual *Np*SRII/*Np*HtrII dimers. SANS experiments allowed us to propose a "tripod"-shaped structural model for trimers of dimers of full-length *Np*SRII/*Np*HtrII in which contacts between dimers occur only at the tips of the cytoplasmic regions, leaving the membrane domains unhinged. In the case of SAXS the change of the salt concentration corresponds to a wide range of the scattering length density (SLD) of the buffer, which would result in a contrast variation simultaneously with the changes of the oligomeric state. Such data would be difficult to treat, also because of a detergent belt for which the contrast variation is a big challenge itself. An electron density of a buffer varies in a range from 0.334 e/Å^3^ (pure H_2_O or D_2_O) to ~ 0.37 e/A^3^ (4 M NaCl in H_2_O), that means the changes of about ~ 11%; electron densities in a protein/detergent system are ~ 0.42, 0.275 and 0.515 e/Å^3^ for a protein, hydrophobic and hydrophilic parts of a detergent belt, respectively. It means that ΔSLD values for different components of detergent/protein complexes have different sign and their ratio critically depends on SLD of the buffer, i.e. on salt concentration. This leads to changes in SAXS I(q) profiles caused by this salt concentration changes. In contrast to SAXS data, in the case of SANS the SLD of the solvent is slightly changed with the increase of the salt concentration [see Table S2a). SLD of a D_2_O buffer changes from 6.404 × 10^–6^ Å^−2^ to 6.187 × 10^–6^ Å^−2^ at the increase of salinity from zero to 4 M (see the section (a) of the Table S2)], that means about 3%. The SLDs of a protein and the hydrophobic and hydrophilic parts of a detergent belt are about 2.1 × 10^–6^ Å^−2^, − 0.4 × 10^–6^ Å^−2^ and 3.9 × 10^–6^ Å^−2^, respectively, i.e. all ΔSLD < 0 and have the same sign. It allows treating SANS data with focusing on a detailed distribution of oligomeric state, which has a more significant influence on a SANS 1D scattering profile than the changes in SLD contrasts at different salt concentrations have.

Our small angle scattering experiments open up prospects for further successful use of this technique for studies of the TCS sensors. Some studies report huge conformational changes of the tertiary structure of the transducer after activation of the photosensor. They are manifested by a change of the diffusion coefficient (and, consequently, of the Stokes radius) of the protein complex and can be explained by unfolding of HAMP domains during signal transfer^52,53^. The changes in the Stokes radius, as well as in the radius of gyration, could be easily detected and characterized using SAS (direct measurements by SAXS). Furthermore, small-angle scattering experiments could provide detailed information about the shape of the molecule, including those arising from changes in tertiary and quaternary structure and/or conformational rearrangements. These changes could, for example, be monitored/characterized via standard SAS experiments with a continuously light-irradiated sample^54^, or using time-resolved SAXS technique^55,56^ applied for the protein solution after the short flash of light. For the particular case studied here there are known mutations of the *Np*SRII (D75N) and in the *Np*HtrII (G83F), which can be used for this purpose^57^ and such experiments may help to obtain detailed structural information concerning the mechanism of activation of *Np*SRII/*Np*HtrII trimer of dimers.

## Methods

### Cloning

*Np*SRII (UniProt ID P42196) and *Np*HtrII (UniProt ID P42259) genes were optimized for expression in *Escherichia coli* using GeneArt services^58^. The Strep-tag II (WSHPQFEK with ENS-linker) and 6xHis-tag (with ENS-linker as well) were added to C-termini of the *Np*SRII and *Np*HtrII respectively. The modified *Np*SRII and *Np*HtrII genes were cloned into pSCodon1.2 vector using NheI-AccIII and NdeI-NheI restriction enzymes (FastDigest series, ThermoFisher, Massachusetts, USA), respectively. The gene of the truncated transducer *Np*HtrII_137_ was obtained from *Np*HtrII by PCR. Positive clones were identified by restriction analysis and confirmed by sequencing. *Np*SRII and *Np*HtrII (or *Np*HtrII_137_) genes were then cloned in tandem into the co-expression vector pET27bmod^59^ via BglII-NotI and NotI-BlpI restriction sites. *E. coli* strain Top10 (Invitrogen, Carlsbad, CA, USA) was used throughout.

### Co-expression of ***Np***SRII and ***Np***HtrII_137_

The non-fused protein complex was co-expressed in *E. coli* cells, strain BL21(DE3) (Stratagene, La Jolla, CA, USA). Briefly, the culture was grown at 37 ℃ in Terrific Broth (TB) media with 100 mM of Na/K-Pi (pH 6.7), 25 mM of ammonium sulfate and 100 mg/L of kanamycin. Expression was induced with 1 mM isopropyl-β-d-galactopyranoside (IPTG), at OD_600_ = 1.6–2.0. Simultaneously, a concentrated solution of all-*trans* retinal in ethanol was added to a final concentration 10 µM retinal. Cells were further cultivated for 4 h at 37 °C.

### Co-expression of *Np*SRII and full-length *Np*HtrII

For SAXS measurements, the non-fused protein complex was co-expressed in *E. coli* strain BL21-AI (Invitrogen, Carlsbad, CA). The culture was grown at 37℃ in TB media with 70 mM of Na/K-Pi (pH 6.7) and 50 mg/L of kanamycin. Expression was induced with the mixture of 0.1% arabinose and 2 mM IPTG, at OD_600_ = 1.6–2.0. Simultaneously, all-*trans* retinal solution in ethanol was added to a final concentration 10 µM, and cells were further cultivated 4 h at 37 °C. For SANS measurements, the non-fused protein complex was co-expressed in *E. coli* strain BL21(DE3). The culture was grown in TB-5052 media^60^ with 100 mM of Na/K-Pi (pH 6.7), 25 mM of ammonium sulfate and 100 mg/L of kanamycin. The cells were incubated at 37 °C until OD_600_ reached 1.0–1.2, when all-trans retinal solution in ethanol was added to final concentration 10 µM, and cells were further cultivated overnight at 20 °C as was described in^61^ for expression of the *Np*SRII.

### Co-purification of ***Np***SRII and full-length ***Np***HtrII (or ***Np***HtrII_137_)

After expression, cells were pelleted in 25 mM Na/Na-Pi buffer (pH 8.0) containing 150 mM NaCl, 1 mM PMSF and cOmplete protease inhibitor cocktail (Roche, Switzerland) and lysed using Microfluidizer M-110P (Microfluidics, Massachusetts, USA). After centrifugation, the pellet was solubilized in 1% of n-dodecyl-β-d-maltopyranoside (DDM). The solubilized protein mixture was then purified via Ni-NTA (HisTrap HP 5 ml column, GE Healthcare, Illinois, USA) and size-exclusion chromatography (Superose 6 10/300 GL column 24 ml, GE Healthcare, USA). A typical gel-filtration profile is shown in Fig. S9A. Laemmli 12% SDS-PAGE of purified *Np*SRII/*Np*HtrII is shown in Fig. S10 (PageRuler Plus Prestained Protein Ladder (Thermo Scientific, Catalog #26619) was used as a molecular weight standard). The protocols for co-expression and co-purification were based on procedures used in^62,63^. The procedure yielded about 5 mg and 1.5 mg per 1 L of cell culture for *Np*SRII/*Np*HtrII_137_ and *Np*SRII/*Np*HtrII, respectively.

### Small-angle X-ray scattering measurements

The majority of SAXS measurements were carried on BM29 beamline (ESRF, Grenoble, France)^64^. All measurements were performed with 100% of beam intensity at a wavelength of 0.9918 Å (12.5 keV). Initial data processing was performed automatically using the EDNA pipeline^65,66^. Additional SAXS experiments were done on the BioSAXS beamline P12 (PETRA III, DESY, Hamburg, Germany)^67^.

For the full-length *Np*SRII/*Np*HtrII and for the truncated complex *Np*SRII/*Np*HtrII_137_, SAXS profiles were obtained for the samples with protein concentrations of 0.57 and 0.78 mg/ml, correspondingly; exposure time was 7 and 10 s, respectively. For all SAXS measurements, peak fractions after gel-filtration were used^68^. See Table S1 for other details of SAXS measurements.

### Small-angle neutron scattering measurements

SANS measurements were performed on the YuMO spectrometer (IBR-2, Dubna, Russia) with two-detector system^69,70^. Raw data were processed with program SAS^71^.

For SANS measurements, *Np*SRII/*Np*HtrII sample was divided after gel filtration into two parts (sample A and B). For each part, gel-filtration was used to replace H_2_O with D_2_O (Fig. S9A). The buffer for sample A contained 150 mM NaCl, 25 mM Na/Na-Pi (pD^+^ = 8.0)^72^, 1 mM EDTA, 0.05% DDM. Sample B was dissolved in 4.0 M NaCl, 100 mM Na/Na-Pi (pD^+^ = 8.0), 1 mM EDTA, 0.05% DDM.

67.5% (v/v) of the stock, containing protein in 150 mM NaCl and 32.5% (v/v) of the stock, containing protein in 4.0 M NaCl were taken to prepare the sample with the *Np*SRII/*Np*HtrII in 1400 mM NaCl; 31.2% (v/v) of the stock, containing protein in 150 mM NaCl and 68.8% (v/v) of the stock, containing protein in 4.0 M NaCl were taken to prepare the sample with the *Np*SRII/*Np*HtrII in 2800 mM NaCl.

The concentrations of the *Np*SRII/*Np*HtrII for the SANS measurements were 0.51, 0.33, 0.31, and 0.40 mg/ml in the buffers containing 150, 1400, 2800, and 4000 mM NaCl, correspondingly. Total exposure time was 2 h for heavy water solutions of the *Np*SRII/*Np*HtrII complex at 150 and 4000 mM NaCl and 3.5 h for solutions at 1400 and 2800 mM NaCl. See Table S2 for other details of SANS measurements.

### Molecular modeling

Initial model of the full-length *Np*SRII/*Np*HtrII dimer was generated from the existing high resolution crystal structures of *T. maritima* MCP, PDB code 2ch7^38^, *E. coli* NarQ receptor, PDB code 5jeq^18^ and truncated *N. pharaonis Np*SRII/*Np*HtrII complex, PDB code 1h2s^24^ for the cytoplasmic domain, HAMP domains and transmembrane region, respectively, using template-based homology modeling in SWISS-MODEL^73^. The missing fragments (primarily, the inter-HAMP region, which was predicted to be an α-helix^74^) were modeled ab initio as ideal helices.

The obtained all-atom model of the full-length *Np*SRII/*Np*HtrII dimer was a subject for further optimization by MD simulation during 100 ns with a number of constraints applied. The regions homological to those resolved by X-ray crystallography were guided towards the initial coordinates by means of harmonic steering forces (k_constr_ = 150 kcal/mol/Å^2^). At each timestep, the RMS distance between the current coordinates and the target structure were computed after aligning the target structure to the current coordinates. The alignment and evaluation of the steering forces was independently done for the transmembrane region, HAMP domains and the cytoplasmic domain. In addition, the inter-HAMP region and the short fragments connecting the TM2 of *Np*HtrII and HAMP1 and AS2 of HAMP2 and the cytoplasmic domain were restrained in α-helical conformation by means of harmonic dihedral restraints.

The initial model for trimer-of-dimers was built by aligning the optimized dimer model to the model of trimeric oligomer of the transmembrane region of *Np*SRII/*Np*HtrII predicted by the SymDock protein–protein docking web-service with the C3 symmetry constraints^75^. This model was further optimized using a 100 ns long MD simulation with the transmembrane region restrained at its initial coordinates and the cytoplasmic domain steered (k_steer_ = 200 kcal/mol/Å^2^) to the homology model of the highly conserved trimeric interface of *E.coli* Tsr receptor resolved by X-ray crystallography, PDB code 1qu7^22^.

Finally, in order to obtain the "tripod"-shaped conformation of the trimer-of-dimer we run another round of steered MD, in which the cytoplasmic tip of the complex was constrained to the crystallographic contacts similar to the previous simulation while the transmembrane regions of individual dimers were gradually repulsed from their joint center-of-masses using the *colvar* feature of NAMD (k_spring_ = 200 kcal/mol/Å^2^). The simulation time of this run was also 100 ns.

All all-atom MD simulations were carried out using NAMD 2.9^76^ and CHARMM27 force field^77^. The simulations were run in NVT ensemble (maintained by the Langevin thermostat, T = 303.15 K) using Generalized Born implicit solvent model (GBIS). The ionic strength in the simulations were set to 4.0 M. The timestep of 2 fs was used. The production simulations were prefaced by energy minimization using the steepest descent (5000 steps). In all simulations of the trimer-of-dimers, the three-fold symmetry was maintained by the symmetry constraint as realized in NAMD 2.9.

### SAS data processing

SAXS and SANS profiles I(q) were processed using ATSAS^78^ and BioXTAS RAW^79^ software suites. The protein concentrations were small, consequently the structural factor influence to scattering curves was negligible^80,81^. For calculation of values of ε, molecular mass, $\overline{\nu }$ and SLD from sequence, programs ProtParam^82^, Peptide Property Calculator^83^ and SLD calculator web (https://sld-calculator.appspot.com/) were used (see Tables S1, S2). Distance distribution functions P(r) and regularized I(q) were obtained using GNOM program, which realizes the method of Indirect-Fourier Transform (IFT)^84^. Values of R_G_ and I(0) (Tables S1, S2) were calculated from P(r) and using Guinier approximations (see Fig. S11). CRYSOL and CRYSON programs were used for evaluating the solution scattering from macromolecules and fitting it to experimental small-angle scattering curves^85,86^. OLIGOMER program^87^ from ATSAS software suite was used for a set of curves calculated for dimers and trimers of dimers using CRYSON to fit an experimental scattering curve from a two-component mixture of dimers and trimers of dimers of the *Np*SRII/*Np*HtrII to validate different variants of their molecular models and to find the volume fractions of each component in the mixture. MEMPROT software^88^ was used to generate pseudo-atomic model of the detergent belt of the transmembrane part of the protein and to fit experimental SAXS curves using a model combined from the detergent belt pseudo-atomic model and protein atomic model. Before MEMPROT running, the center of the transmembrane part of the protein was placed at the origin (zero) and direction of the normal vector to the membrane plane was set along the z-axis using PPM web server^89^. The MEMPROT settings included CRYSOL3 option and protein surface algorithm 2. See Supplementary Information (Tables S1, S2) for other details of SAS data treatment^90^.

### Pseudo-atomic model of the detergent belt

Pseudo-atoms CH_3_ и NH_3_ generated by MEMPROT software simulate 9/97 of the X-ray scattering length of hydrophobic tail (C_12_H_25_) and 10/181 of the X-ray scattering length of hydrophilic head (C_12_H_21_O_11_) of DDM molecule correspondingly, proportionally to their numbers of electrons. To calculate theoretical SANS curves of the protein with the DDM belt, pseudo-atoms NH_3_ generated by MEMPROT were renamed to CH_3_, and perdeuturation parameter in CRYSON settings were set to 0.1058 and 0.3903 for chains corresponding to hydrophobic and hydrophilic parts of the DDM belt respectively. Described procedure provides neutron scattering lengths − 1.27 fm (9/97 of C_12_H_25_) and 7.62 fm (10/181 of C_12_H_14_D_7_O_11_) for pseudo-atoms related to hydrophobic and hydrophilic parts of the detergent belt respectively, which corresponds to the same scattering length fractions as they are in case of X-ray scattering length. Here, it is taken into account that the DDM head has seven hydrogens exchanging to deuterium in D_2_O. These procedure provides neutron SLD values of − 0.388 × 10^–6^ Å^−2^ and 3.92 × 10^–6^ Å^−2^ for the hydrophobic core and hydrophilic face of the detergent belt, respectively.

### Fitting of the SAXS profile of the full-length complex using the model of the flexible hinges

Firstly, SAXS data for the full-length complex *Np*SRII/*Np*HtrII (Fig. 2A, top) were approximated with the solution scattering evaluated from the atomistic model of the "straight" *Np*SRII/*Np*HtrII dimer with pseudo-atomic model of the detergent belt generated by MEMPROT software^88^, and χ^2^ of the fit was 5.1. Secondly, we generated modified atomic models of the *Np*SRII/*Np*HtrII dimer with the bends at the HAMP1-, HAMP2- and Gly- hinges from − 90° to 90° each (with the step of 30°), and add pseudo-atoms imitating detergent belt obtained on previous step to them. Thirdly, we evaluated theoretical SAXS profiles from the modified *Np*SRII/*Np*HtrII dimer models with the detergent belt using CRYSOL3 software^78^. Fourthly, we approximated experimental SAXS profile for the full-length *Np*SRII/*Np*HtrII as a combination of the scattering profiles from the modified *Np*SRII/*Np*HtrII dimer models with the detergent belt using Tikhonov regularization method (see Text document S1 for the details). For fitting of this data, Wolfram Mathematica software^91^ was used. Analogous analysis of the protein polydispersity based on SAS data was realized in works^92,93^. SAXS could be used in studying of highly polydisperse macromolecules, making it possible to obtain data that are in good agreement with other structural methods such as electron microscopy^94^.

### UV–VIS spectroscopy

Absorption spectra for the protein solutions were acquired by using a UV-2450 UV–VIS Spectrophotometer (Shimadzu, Kyoto, Japan). Protein solutions were placed in a 1 mm quartz cell (Weiju, Lianyungang, China). To estimate concentration of the photoactive complex, the value ε^498nm^ = 45,500 M^−1^ cm^−1^ of the *Np*SRII extinction coefficient was used, and the sensory rhodopsin and its transducer were assumed to be equimolar. Absorption spectrum of the sample of the full-length *Np*SRII/*Np*HtrII complex in D_2_O buffer with 150 mM NaCl is shown in Fig. S9B.

### CD spectroscopy

CD spectra were acquired by using a J-1100 CD Spectrometer (Jasco, Easton, MD, USA). For CD measurements, the same samples were used as for SANS measurements. Protein solutions were placed in a 1 mm quartz cell (Weiju, Lianyungang, China). All spectra were recorded with a bandwidth of 1.0 nm, scan speed of 50 nm/min, and digital integration time of 1.0 s. For the protein at 150 and 4000 mM NaCl, five accumulations were averaged for each spectrum, for 1400 and 2800 mM of NaCl, averaging of eight accumulations was done. The quantification of secondary structures was analyzed by Dichroweb^95,96^ using K2D program^97^.

## Supplementary Information

Supplementary Information 1.

## Data Availability

The SAS data were deposited with SASBDB (http://sasbdb.org). SAXS data deposited with accession codes SASDKZ6 and SASDK27 for the *Np*SRII/*Np*HtrII_137_ and the full-length *Np*SRII/*Np*HtrII at 150 mM NaCl, correspondingly. SANS data deposited with accession codes SASDK37, SASDK47, SASDK57, and SASDK67 for the full-length *Np*SRII/*Np*HtrII at 0.15 M, 1.4 M, 2.8 M and 4.0 M, respectively. Detailed data validation metrics related to SASBDB depositions are placed in the Table S3. PBD files for molecular models of *N**p*SRII/*N**p*HtrII_1__3__7_ dimer and full-length *N**p*SRII/*N**p*HtrII dimer and trimer of dimers are available in the SASBDB depositions SASDKZ6, SASDK27, and SASDK67, respectively. Other data supporting the findings of this manuscript are available from the corresponding author upon reasonable request.
