# Supplementary material for: Molecular model of a sensor of two-component signaling system
Source: Sci Rep. 2021 May 24;11:10774. doi: 10.1038/s41598-021-89613-6 (PMC8144572; doi:10.1038/s41598-021-89613-6)
Supplement: Supplementary file 1 — Supplementary Information 1. [file 41598_2021_89613_MOESM1_ESM.docx]

**Molecular model of a sensor of two-component signaling system**

Yury L. Ryzhykau^†1^, Philipp S. Orekhov^†1^, Maksim I. Rulev^†2,3,4^, Alexey V. Vlasov^1,5^, Igor A. Melnikov^2^, Dmytro A. Volkov^3,4^, Mikhail Yu. Nikolaev^1,3^, Dmitrii V. Zabelskii^1,3,4^, Tatiana N. Murugova^1,6^, Vladimir V. Chupin^1^, Andrey V. Rogachev^1,6^, Andrey Yu. Gruzinov^7^, Dmitri I. Svergun^7^, Martha E. Brennich^8^, Ivan Yu. Gushchin^1^, Montserrat Soler-Lopez^2^, Arne Bothe^9^, Georg Büldt^1^, Gordon Leonard^2^, Martin Engelhard^9^, Alexander I. Kuklin^1,6^,*, and Valentin I. Gordeliy^1,3,4,10^,*

^1^Research Center for Molecular Mechanisms of Aging and Age-Related Diseases, Moscow Institute of Physics and Technology, 141700 Dolgoprudny, Russia

^2^Structural Biology Group, European Synchrotron Radiation Facility, 71 Avenue des Martyrs, 38000 Grenoble, France

^3^Institute of Biological Information Processing (IBI-7: Structural Biochemistry), Forschungszentrum Jülich, 52425 Jülich, Germany

^4^JuStruct: Jülich Center for Structural Biology, Forschungszentrum Jülich, 52428 Jülich, Germany

^5^Institute of Crystallography, University of Aachen (RWTH), Jaegerstrasse 17-19, 52056 Aachen, Germany

^6^Frank Laboratory of Neutron Physics, Joint Institute for Nuclear Research, 141980 Dubna, Russia

^7^European Molecular Biology Laboratory, Hamburg Unit, 22607 Hamburg, Germany

^8^Synchrotron Crystallography Team, EMBL Grenoble Outstation, 71 Avenue des Martyrs, 38042 Grenoble, France

^9^Department Structural Biochemistry, Max Planck Institute of Molecular Physiology, 44227 Dortmund, Germany

^10^Institut de Biologie Structurale Jean-Pierre Ebel, Université Grenoble Alpes–Commissariat à l'Energie Atomique et aux Energies Alternatives–CNRS, F-38027 Grenoble, France

*To whom correspondence may be addressed. Email: kuklin@nf.jinr.ru, valentin.gordeliy@ibs.fr.

^†^These authors contributed equally to this work.

**Supplementary Information**


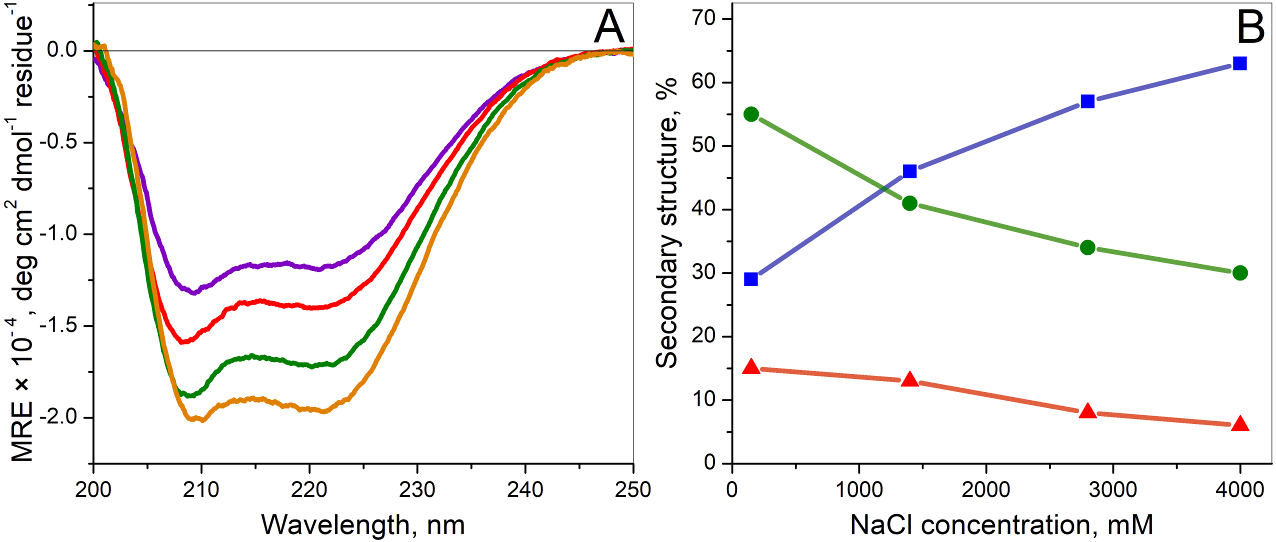


**Figure S1.** Effect of the NaCl molarity on the secondary structure of the full-length *Np*SRII/*Np*HtrII complex. A: far-UV CD spectra (in Mean Residue Ellipticity (MRE) units) of the *Np*SRII/*Np*HtrII complex at 150, 1400, 2800 and 4000 mM NaCl (purple, red, green and orange curves respectively). B: deduced secondary structure content versus NaCl molarity (blue, red and green curves corresponds to helix, sheet and random coil respectively).


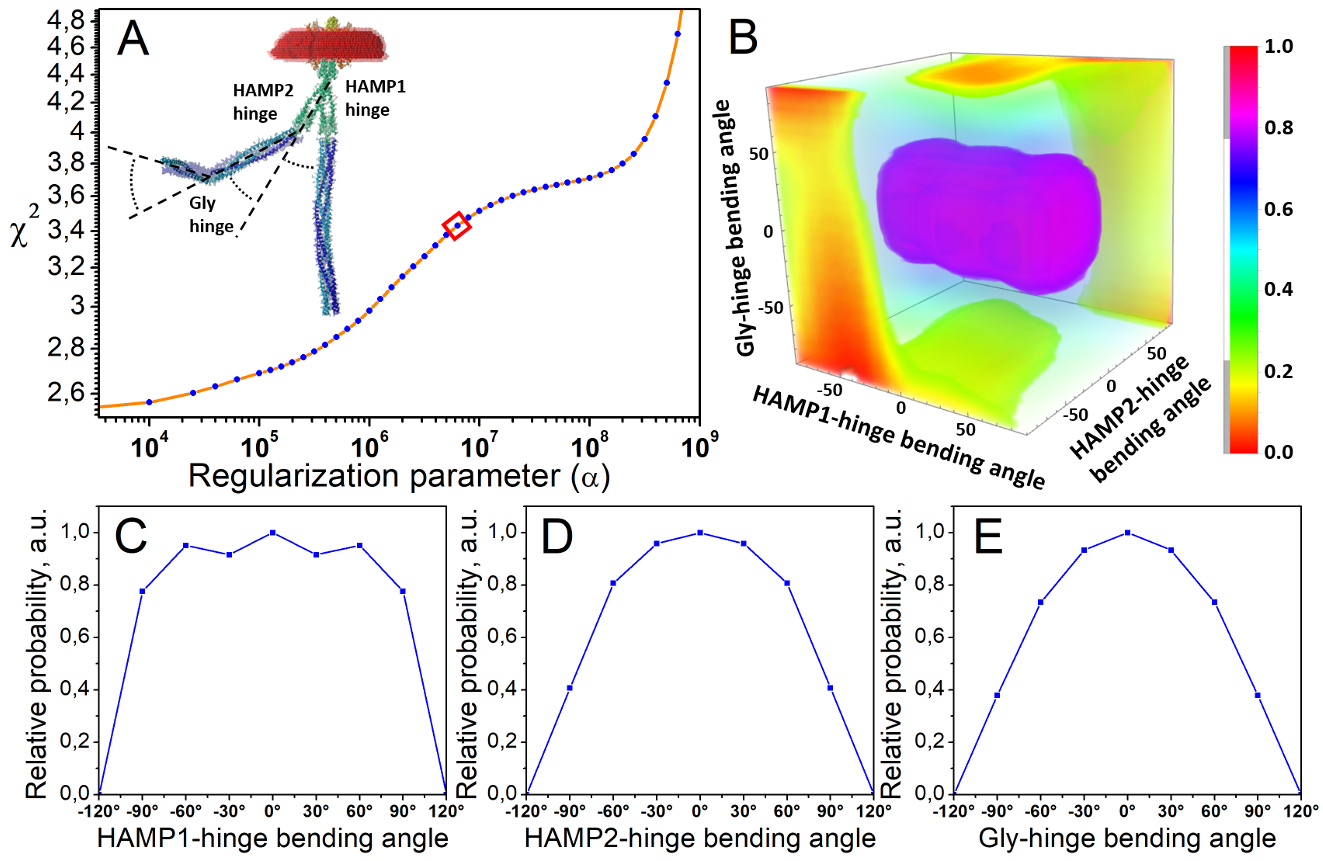


**Figure S2.** Results of fit of SAXS data obtained for *Np*SRII/*Np*HtrII at 150 mM NaCl. A: dependence of chi-square on logarithm of the regularization parameter α (see Text document S1 for the details). Chosen point corresponding to the inflexion point is marked by the red square. Comparison of the "straight" *Np*SRII/*Np*HtrII dimer and dimer bent on HAMP1, HAMP2 and Gly hinges is shown in the upper left corner. B: three-dimensional representation of the probability distribution function of the *Np*SRII/*Np*HtrII dimer to be in conformation with the corresponding bending angles of HAMP1-, HAMP2-, and Gly- hinges (see Fig. 2A (*top*)). C: relative probability of the *Np*SRII/*Np*HtrII dimer to be in conformation with corresponding bend at HAMP1-hinge (obtained by the averaging over the bending angles of HAMP2-, and Gly- hinges of the probability distribution function represented on B). D and E: the analogous dependency as shown on C, obtained for bends at HAMP2-hinge and Gly-hinge, correspondingly. The root mean square deviations from the zero value are equal to 58°, 51°, and 51° for bending angles of HAMP1-, HAMP2-, and Gly- hinges, correspondingly.


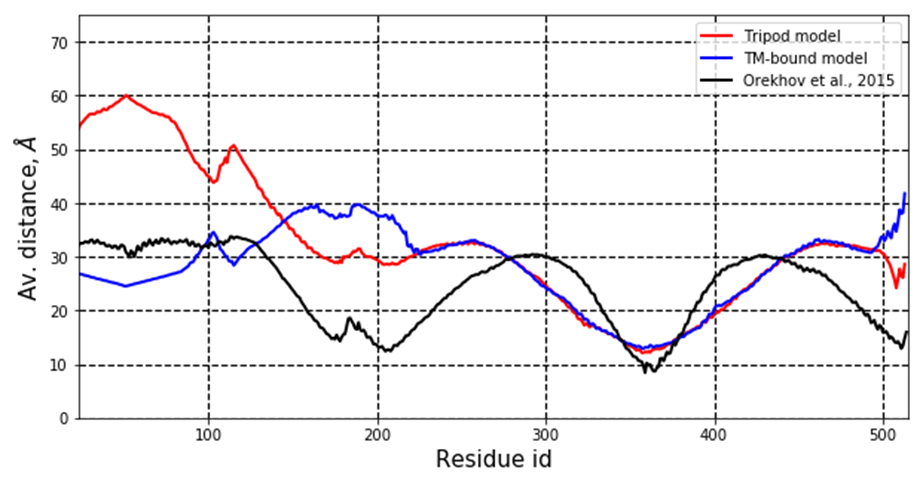


**Figure S3.** The average distances between individual *Np*HtrII dimers and the trimeric axis. Distances calculated for the optimized high-resolution molecular models of the "tripod"-shaped (red) and the transmembrane-bound (blue) trimer of dimers, and the coarse-grained molecular model obtained in (Orekhov, P. S. *et al.* *PLOS Comput. Biol.* **11**, e1004561 (2015)) (black).


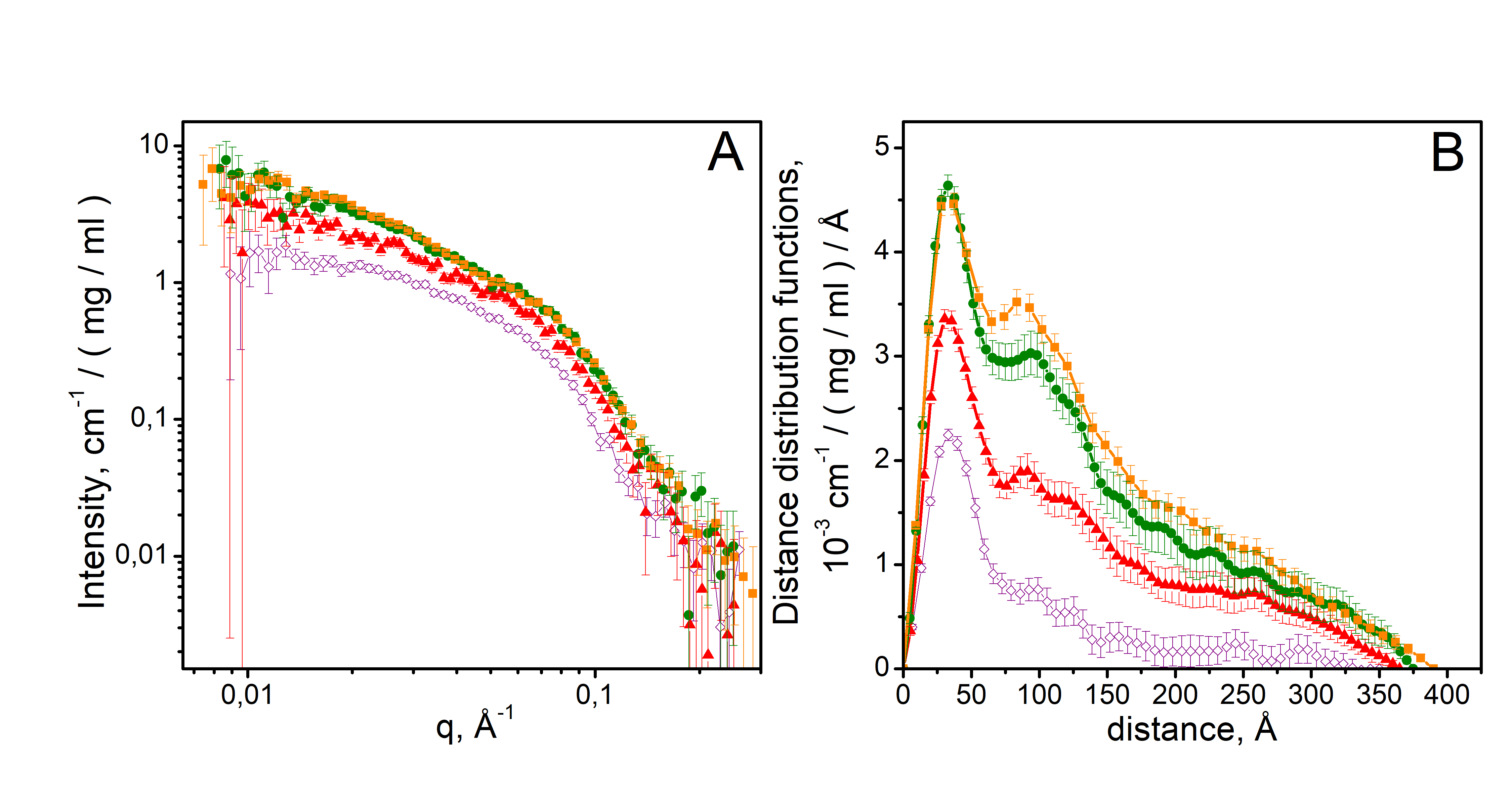


**Figure S4.** Small-angle neutron scattering curves for the *Np*SRII/*Np*HtrII and corresponding distance distribution functions. A: SANS experimental data obtained with the protein *Np*SRII/*Np*HtrII complex in D_2_O solutions with 0.15, 1.4, 2.8 and 4.0 M NaCl (purple hollow rhombus, red triangles, green circles and orange squares respectively). B: distance distribution functions calculated from SANS experimental data shown at part A (with the same designations).


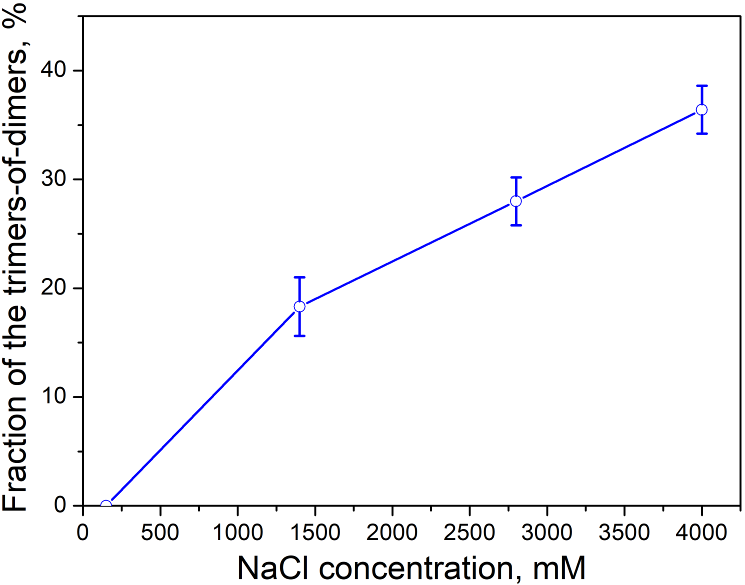


**Figure S5.** Weight fraction of the trimers of dimers of the full-length *Np*SRII/*Np*HtrII complex versus NaCl molarity. Fractions were calculated by fitting of SANS data for the protein at 1400, 2800 and 4000 mM NaCl with combination of theoretical SAS curves of the dimeric model and "tripod"-shaped model of the trimer of dimers using OLIGOMER program from ATSAS software suite. As neutron scattering data obtain for the protein at low salt was fitted well by the model of the dimer (Fig. 1A (*bottom*)), fraction of the trimers-of-dimers at 150 mM NaCl assumed to be zero.


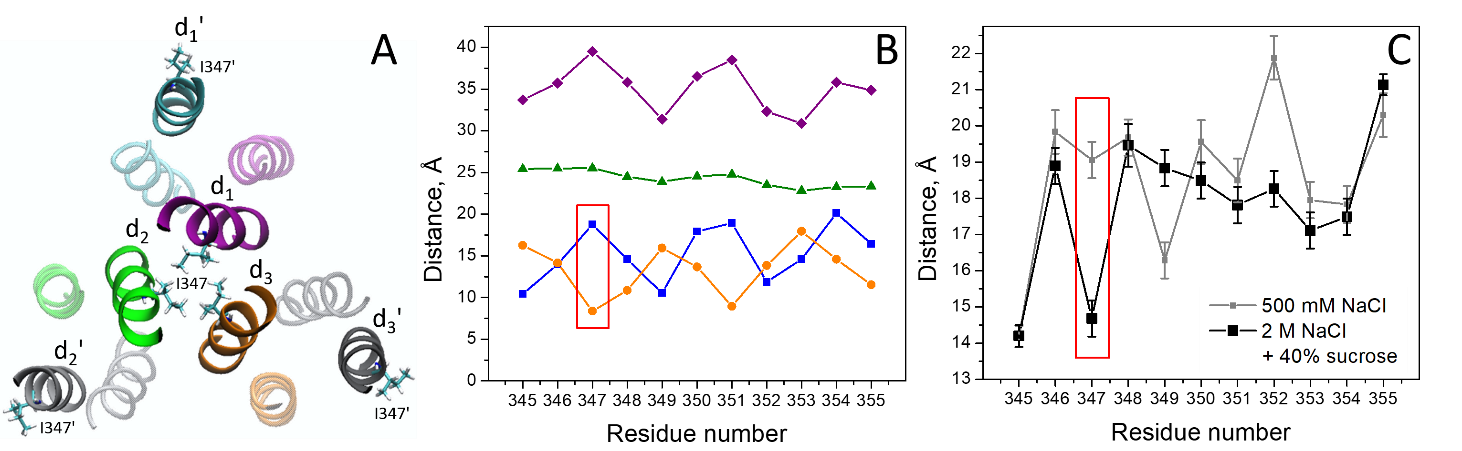


**Figure S6.** Comparison of distances between Cα atoms of 345-355 residues in the "tripod"-shaped molecular model of the *Np*SRII/*Np*HtrII trimer of dimer with interspin distances for spin-labeled residues 345-355 obtained in work (Orban-Glaß, I. *et al.* *Biochemistry.* **54(2)**, 349–362 (2015)). A: representation of the *Np*HtrII tip region depicting hydrophobic contacts between I347. *Np*HtrII dimers are marked as d_1_-d_1_', d_2_-d_2_' and d_3_‑d_3_'. Individual polypeptide chains are colored differently. Regions 345-355 a. a. are shown as opaque, their antiparallel regions are shown as transparent. B: Mean inter-Cα distances obtained from the "tripod"-shaped molecular model. Blue squares correspond to d_1_-d_1_', d_2_-d_2_' and d_3_-d_3_' distances; orange circles correspond to d_1_-d_2_, d_1_-d_3_ and d_2_-d_3_ distances; green triangles correspond to d_1_-d_2_', d_1_-d_3_', d_2_-d_1_' , d_2_-d_3_' , d_3_-d_1_' and d_3_-d_2_' distances; purple rhombus correspond to d_1_'-d_2_', d_1_'-d_3_' and d_2_'-d_3_' distances. C: Mean interspin distances obtained from dipolar broadened EPR powder spectra. Data reconstructed from (Orban-Glaß, I. *et al.* *Biochemistry.* **54(2)**, 349–362 (2015)).

**
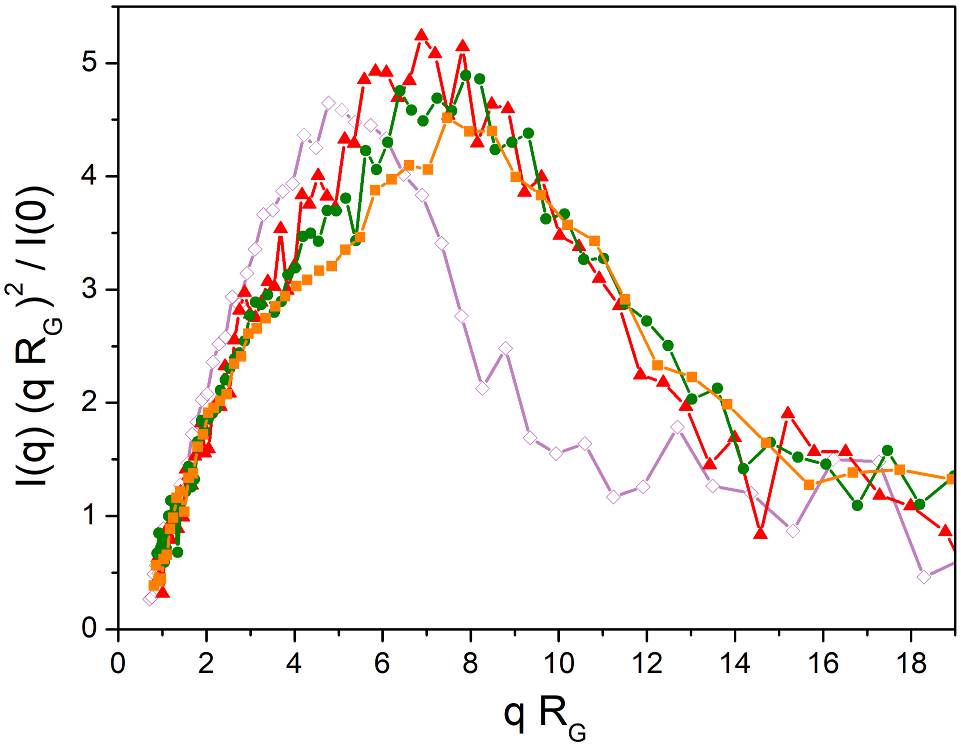
**

**Figure S7.** Normalized Kratky plots obtained from SANS data shown in Fig. S2A (with the same designations). I(0) and R_G_ values were calculated from *P(r)* (see Fig. S3B and Tab. S2 *(d)*).


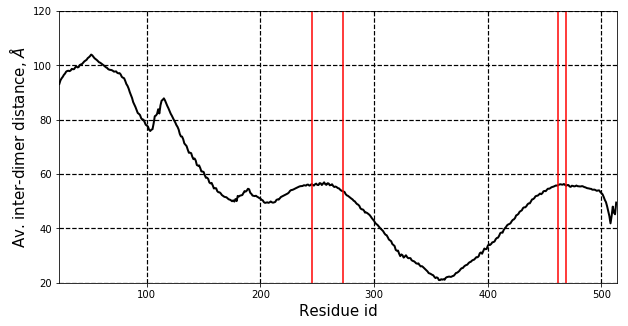


**Figure S8.** The average inter-dimer distances between individual *Np*HtrII dimers of the optimized high-resolution molecular model of the "tripod"-shaped trimers. Putative sites of methylation/demethylation are indicated by the red lines.

**
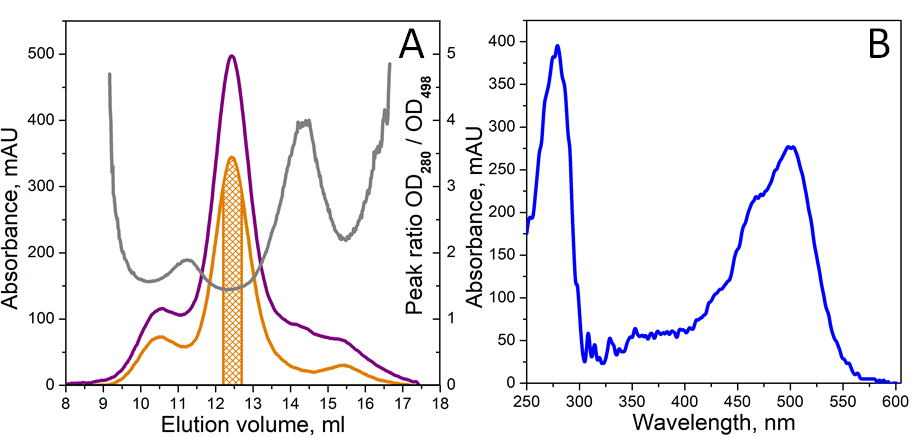
**

**Figure S9.** Purification and characterization of the *Np*SRII/*Np*HtrII. A: gel-filtration profile of the *Np*SRII/*Np*HtrII in D_2_O at 150 mM NaCl. Absorbance at 280 nm is shown in purple; absorbance at 498 nm is shown in orange, and peak ratio OD_280_/OD_498_ is shown in grey. Peak fraction with the minimum peak ratio value were taken for further analysis (shown as the filled area under 498 nm absorbance curve). B: absorption spectrum of the *Np*SRII/*Np*HtrII complex in D_2_O-buffer at 150 mM NaCl (optical density recalculated to path length 10 mm).


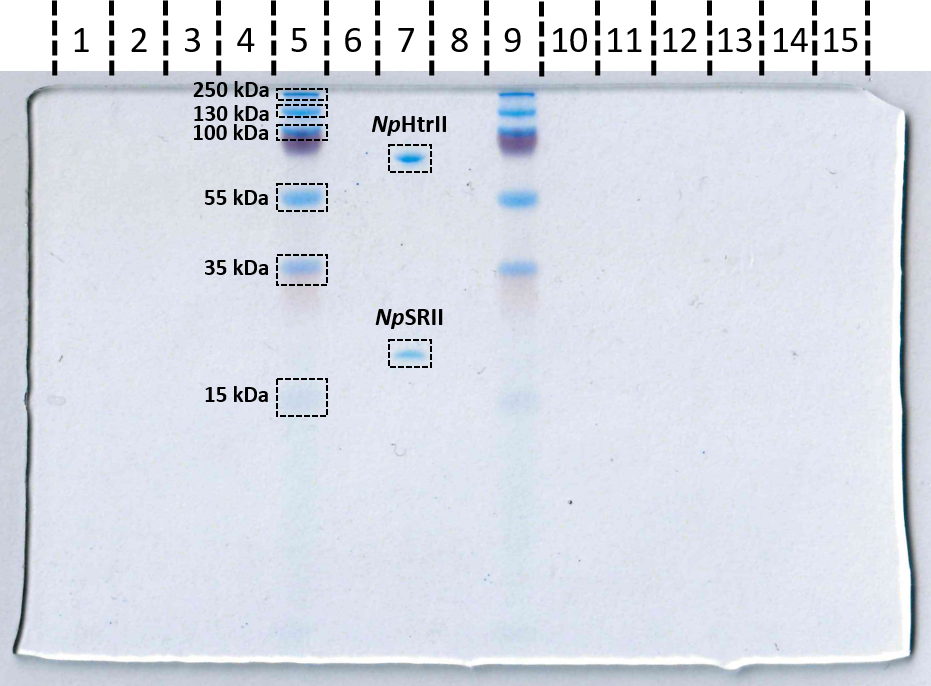


**Figure S10.** Laemmli SDS-PAGE of the *Np*SRII/*Np*HtrII in 12% gel. Wells #5 and #9 correspond to PageRuler Plus Prestained Protein Ladder (Thermo Scientific), blue bands in which correspond to molecular weights of 15, 35, 55, 100, 130 and 250 kDa. Well #7 corresponds to the fraction of the *Np*SRII/*Np*HtrII complex after gel-filtration. The expected molecular weights of *Np*SRII and *Np*HtrII are 26.7 and 57.8 kDa, respectively.


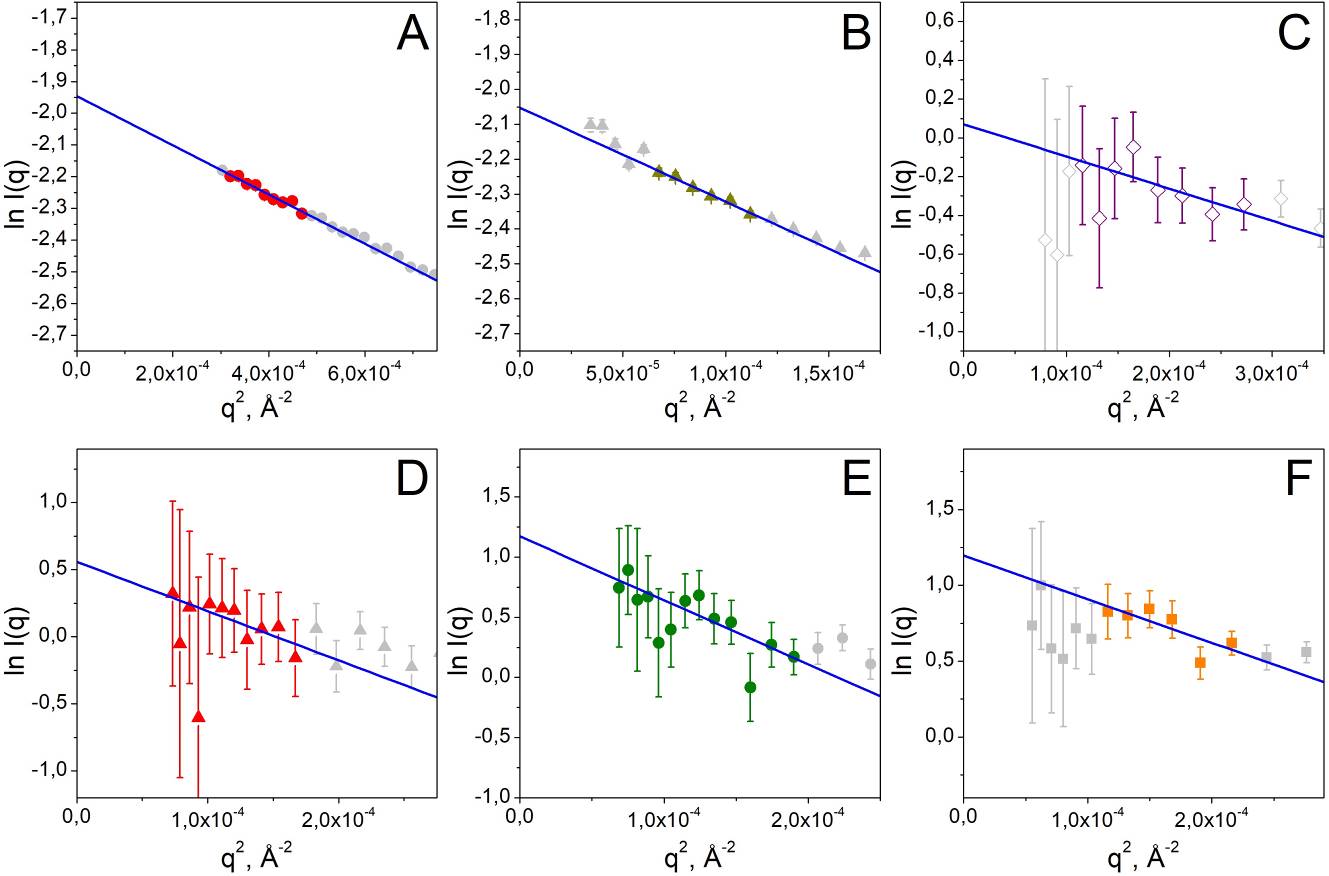


**Figure S11.** Guinier approximations made for SAXS data obtained for truncated *Np*SRII/*Np*HtrII_137_ and full-length *Np*SRII/*Np*HtrII (A and B, respectively) at 150 mM NaCl and SANS data obtained for *Np*SRII/*Np*HtrII at 150, 1400, 2800 and 4000 mM NaCl (C, D, E and F, respectively).

**Text document S1.** Optimization of the fit of the SAXS data using Tikhonov regularization.

For the fitting of SAXS data obtained for the *Np*SRII/*Np*HtrII at 150 mM NaCl as a combination of the modified *Np*SRII/*Np*HtrII dimers with the detergent belt (see Materials and Methods), minimization procedure were done using Wolfram Mathematica software (Quadratic Optimization function) for the following expression:

| $\text{χ}_{\text{SAXS}}^{2}\text{+}\text{ }\text{Ω}\left( \boldsymbol{w} \right)$ | (1) |
| --- | --- |

The first term of the expression (1) is the normalized chi-square of the fit:

| $\text{χ}_{\text{SAXS}}^{2}=\frac{1}{N_{exp}}\cdot\sum_{n=1}^{N_{exp}} \left( \frac{I_{n}-I_{theor}\left( q_{n} \right)}{\sigma_{n}} \right)^{2}$ | (2) |
| --- | --- |

where $N_{exp}$ is a number of experimental points taken for fitting, {$q_{n}$*, $I_{n}$,*$\sigma_{n}$} is a set of the experimental data. Theoretical SAXS profile is given by the following expression:

| $I_{theor}\left( q \right)=\sum_{i} \sum_{j} \sum_{k} w_{i,j,k}\cdot I_{dimer}\left( q,\varphi_{HAMP1}^{i},\varphi_{HAMP2}^{j},\varphi_{Gly}^{k} \right)+cst$ | (3) |
| --- | --- |

where $w_{i,j,k}$ corresponds to the probability of the *Np*SRII/*Np*HtrII dimer to be in conformation with bends of HAMP1-, HAMP2-, and Gly- hinges equal to  $\varphi_{HAMP1}^{i}$, $\varphi_{HAMP2}^{j}$, and $\varphi_{Gly}^{k}$, respectively. The corresponding theoretical solution scattering profile from the *Np*SRII/*Np*HtrII dimer marked as $I_{dimer}\left( q,\varphi_{HAMP1}^{i},\varphi_{HAMP2}^{j},\varphi_{Gly}^{k} \right)$. The additional constant $cst$ accounts for possible systematic errors due to mismatched buffers in the experimental data. In general, the tensor $\boldsymbol{w}$ is a discrete representation of the probability density function $w\left( \varphi_{HAMP1},\varphi_{HAMP2},\varphi_{Gly} \right)$. The condition $w\left( \varphi_{HAMP1},\varphi_{HAMP2},\varphi_{Gly} \right)=w\left( -\varphi_{HAMP1},{-\varphi}_{HAMP2},{-\varphi}_{Gly} \right)$ corresponds to the P2 symmetry expected for the *Np*SRII/*Np*HtrII dimer. As probability is a non-negative value, the condition $w_{i,j,k}\geq0$ was used.

The role of the second term in the expression (1) is to use Tikhonov regularization to avoid overfitting. It is given by the following quadratic form over the tensor $\boldsymbol{w}$:

| $\text{Ω}\left( \boldsymbol{w} \right)=\alpha\cdot\left( \sum_{i} \sum_{j} \sum_{k=2}^{N_{k}-1} \left( w_{i,j,k+1}-w_{i,j,k} \right)^{2}+\sum_{i} \sum_{j=1}^{N_{j}-1} \sum_{k} \left( w_{i,j+1,k}-w_{i,j,k} \right)^{2}++\sum_{i=1}^{N_{i}-1} \sum_{j} \sum_{k} \left( w_{i+1,j,k}-w_{i,j,k} \right)^{2}+\sum_{i} \sum_{j} \left( w_{i,j,1}^{2}+w_{i,j,N_{k}}^{2} \right)++\sum_{i} \sum_{k} \left( w_{i,1,k}^{2}+w_{i,N_{j},k}^{2} \right)+\sum_{j} \sum_{k} \left( w_{1,j,k}^{2}+w_{N_{i},j,k}^{2} \right) \right)$ | (4) |
| --- | --- |

where *α* is a regularization parameter.

Minimization of the first three terms in the expression (4) provides sufficient smoothness of the desired probability density function. We assume that outside the range of the considered angles, the probability density function tends to zero, that, in combination with the smoothness condition, imposes additional boundary conditions, which are provided by minimization of the last three terms of the expression (4).

The regularization parameter *α* is responsible for the degree of the smoothness. At the lowest values of *α*, the fit is the best and the value of the $\text{χ}_{\text{SAXS}}^{2}$ is minimum, however, the solution is not smooth enough. At very high values ​​of the regularization parameter, the solution is quite smooth, but the fit quality decreases, which results in a strong increase in the chi-square value. The good compromise between a chi-square minimization and smoothness optimization could be found near the inflection point of the dependence of the chi-square on the logarithm of the optimization parameter (see Fig. S2A).

**Text document S2.** Sequence alignment of Kinase Control Modules of the *Hs*HtrII (Uniprot ID Q9HP81; 469‑744 residues) and the *Np*HtrII (Uniprot ID P42259; 229‑504 residues).

Sequences have 41.7% identity and 64.1% similarity. Putative methylation sites (D485‑E486, E513‑E514, D702‑E703 and Q709‑E710 in case of *Hs*HtrII and Q245‑N246, E273‑E274, E462‑D463 and E469‑Q470 in case of *Np*HtrII) are marked by green.

*Hs*HtrII 469 DAGDAIDAGTDVSTAVDEISDGATEQTDRLHEVAGEVDDLSASAEEVAET 518

:|...::|..||:.||..|||.|.:||:.:.::|.|:||:||:.||||.:

*Np*HtrII 229 NAESVMEASEDVNRAVQNISDAAGDQTETVQQIALEMDDVSATTEEVAAS 278

*Hs*HtrII 519 VASLADTAGQAASAVDDGRQATEDAVETMDDVADDAEAAADAMDALDSEM 568

...:|.||.|||...:.||:..|.|:..|::|....|.|..:|:.|:.::

*Np*HtrII 279 ADDIAKTARQAAETGEAGRETAETAITEMNEVESRTEQAVASMEELNEDV 328

*Hs*HtrII 569 ADIGEIVDVIADIADQTNMLALNASIEAARTGADGDGFAVVADEVKTLAE 618

.:|||:.::|||||:|||:|||||||||||...:.:||||||||||.|||

*Np*HtrII 329 REIGEVSEMIADIAEQTNILALNASIEAARADGNSEGFAVVADEVKALAE 378

*Hs*HtrII 619 ESRDAAEDIESRLLALQGQVSDVADEMRATSDTVSDGRATVGDAATALDD 200

|::.|.|:|:..:..:|.:.....|::|.|||.||:|..||.|...||:.

*Np*HtrII 379 ETKAATEEIDDLIGTVQDRTQTTVDDIRETSDQVSEGVETVEDTVDALER 428

*Hs*HtrII 669 VVSFVADTDTAAGEIRAATDRQAHAASRVASAVDEVAGISQETAAQATAV 718

:|..|..|:....||..:||.||.||.:..:.|:::|..|::||:.|...

*Hs*HtrII 429 IVDSVERTNDGIQEINQSTDAQADAAQKATTMVEDMAATSEQTASDAETA 478

*Hs*HtrII 719 ADSAATQTDTLSSVDDAAADLADRA 744

|::..||.:::..|.|....|:::|

*Np*HtrII 479 AETTETQAESVKEVFDLIDGLSEQA 504

**Table S1.** SAXS experimental details and data evaluation summary.

| ***(a)* Sample details^1^** | | | | |  |
| --- | --- | --- | --- | --- | --- |
|  | *Np*SRII/*Np*HtrII_137_ | | *Np*SRII/*Np*HtrII | |  |
| Description of sequence | The complex of Sensory rhodopsin II (UniProt ID P42196) with its cognate transducer (UniProt ID P42259) from *N. pharaonis* | | | |  |
|  | Truncated transducer (1-137 a.a.) | | Full-length transducer | |  |
| Extinction coefficient ε (M^‑1^ cm^‑1^) | 118 720 (280nm); 91 000 (498 nm) | | | |  |
| Partial specific volume $\bar{\nu}$ (cm^3^/g) | 0.728635 | | 0.642196 | |  |
| Mean solute and solvent SLD (10^‑6^ Å^‑2^) | 12.516, 9.465 | | 14.165, 9.465 | |  |
| Mean scattering contrast $\Delta\bar{\rho}$ (10^‑6^ Å^‑2^) | 3.051 | | 4.700 | |  |
| Molecular mass (Da) | 83386.88 | | 168969.82 | |  |
| Sample concentration (mg/ml) | 0.78 | | 0.57 | |  |
| Solvent composition | 150 mM NaCl, 25 mM Na/Na-Pi, 1.0 mM EDTA, 0.05% DDM, pH 8.0 | | | |  |
| **(*b*) SAS data collection parameters** | | | | |  |
| Instrument | ESRF BM29 | | | |  |
| Wavelength (Å) | 0.9918 | | | |  |
| Beam geometry | Size: 700 × 700 µm^2^; Sample-to-detector distance: 2.864 m | | | |  |
| Sample configuration | 1.8 mm-diameter quartz capillary | | | |  |
| *q*-measurement range (Å^−1^) | 0.004 – 0.5 | | | |  |
| Absolute scaling method | Comparison with scattering from pure H_2_O | | | |  |
| Basis for normalization to constant counts | To transmitted intensity by direct beam counter | | | |  |
| Exposure time | 10 sec | | 7 sec | |  |
| Sample temperature (°C) | 20 | | | |  |
| **(*c*) Software employed for SAS data reduction, analysis and interpretation** | | | | |  |
| SAS data averaging and subtraction | PRIMUSqt from ATSAS 2.8.4 | | | |  |
| Calculation of ε from sequence | ProtParam: <https://web.expasy.org/protparam/> | | | |  |
| Calculation of $\bar{\nu}$ values from chemical composition | Peptide Property Calculator: <http://biotools.nubic.northwestern.edu/proteincalc.html> | | | |  |
| Calculation of $\bar{\rho}$ values from chemical composition | SLD calculator web: <https://sld-calculator.appspot.com/> | | | |  |
| Guinier, *P*(*r*) | GNOM from ATSAS | | | |  |
| Atomic structure modelling | MEMPROT 2.2; CRYSOL 3.0 | | | |  |
| Molecular graphics | VMD 1.9.3 | | | |  |
| **(*d*) Structural parameters** | | | | |  |
| Guinier analysis |  | |  | |  |
| I(0) (cm^-1^) | 0.143 ± 0.002 | | 0.128 ± 0.003 | |  |
| R_g_ (Å) | 45.2 ± 1.84 | | 89.9 ± 4.6 | |  |
| q R_G_ -range | 0.7 – 0.87 | | 0.74 – 0.95 | |  |
| *P(r)* analysis |  |  |  |  |  |
| I(0) (cm^-1^) | 0.138 ± 0.004 | | 0.132 ± 0.011 | |  |
| R_G_ (Å) | 45.74 ± 0.12 | | 98.4 ± 1.5 | |  |
| d_max_ (Å) | 140 | | 389 | |  |
| q-range (Å^−1^) | 0.0151 – 0.350 | | 0.00586 – 0.350 | |  |
| q_min_ d_max_ / π | 0.673 | | 0.725 | |  |
| Total quality estimate (GNOM) | 0.858 | | 0.604 | |  |
| ***(e)* Atomistic modelling** | | | | |  |
|  | *Np*SRII/*Np*HtrII_137_ | | *Np*SRII/*Np*HtrII | |  |
| Method | MEMPROT; CRYSOL 3.0 | | | |  |
| q-range for fitting | 0.0151 – 0.350 | | 0.00586 – 0.350 | |  |
| Any measures of model precision | Atomic models of the *Np*SRII/*Np*HtrII dimers were generated as described in Materials and Methods. Pseudo-atomic detergent belt model was generated using MEMPROT program.  Quadratic optimization function of Wolfram Mathematica 12.0 was used to fit an experimental scattering curve from a mixture of *Np*SRII/*Np*HtrII dimers bended at flexible hinges. | | | |  |
| Background subtraction (cm^-1^) | 0.055 × 10^-3^ | | "Straight" *Np*HtrII: 0.055 × 10^-3^  Superposition of dimers with bended transducers: 0.141 × 10^-3^ | |  |
| χ^2^ value; *P* value (from CorMap) | 1.524; 0.000 | | "Straight" *Np*HtrII: 5.149; 0.000  Superposition of dimers with bended transducers: 3.435; 0.000 | |  |
| ***(f)* Data and model deposition IDs** | | | | |  |
|  | *Np*SRII/*Np*HtrII_137_ | | *Np*SRII/*Np*HtrII | |  |
|  | SASDKZ6 | | SASDK27 | |  |

^1^ Parameters were calculated for the dimer of the *Np*SRII/*Np*HtrII complex without detergent belt.

**Table S2.** SANS experimental details and data evaluation summary.

| ***(a)* Sample details^1^** | | | | |  |
| --- | --- | --- | --- | --- | --- |
|  | *Np*SRII/*Np*HtrII  at 0.15 M NaCl | *Np*SRII/*Np*HtrII  at 1.4 M NaCl | *Np*SRII/*Np*HtrII  at 2.8 M NaCl | *Np*SRII/*Np*HtrII  at 4 M NaCl |  |
| Description of sequence | Dimer of the complex of Sensory rhodopsin II (UniProt ID P42196) with its cognate transducer (UniProt ID P42259) from *N. pharaonis* | | | |  |
| Extinction coefficient ε (M^‑1^ cm^‑1^) | 118 720 (280nm), 91 000 (498 nm) | | | |  |
| Partial specific volume $\bar{\nu}$ (cm^3^/g) | 0.642196 | | | |  |
| Mean solute and solvent SLD (10^‑6^ Å^‑2^)**^2^** | 2.1235; 6.404 | 2.1235; 6.334 | 2.1235; 6.259 | 2.1235; 6.187 |  |
| Mean scattering contrast $\Delta\bar{\rho}$ (10^‑6^ Å^‑2^)**^2^** | -4.281 | -4.211 | -4.136 | -4.064 |  |
| Molecular mass (Da)**^2^** | 168969.82 | | | |  |
| Sample concentration (mg/ml) | 0.51 | 0.33 | 0.31 | 0.40 |  |
| Solvent composition | 150 mM NaCl,  25 mM Na/NaPi | 1400 mM NaCl,  49 mM Na/NaPi | 2800 mM NaCl,  77 mM Na/NaPi | 4000 mM NaCl,  0.1 M Na/NaPi |  |
|  | 1.0 mM EDTA, 0.05% DDM, pD^+^ = 8.0 (D_2_O buffers) | | | |  |
| **(*b*) SAS data collection parameters** | | | | |  |
| Instrument | YuMO, IBR-2, FLNP, JINR (Dubna, Russia) | | | |  |
| Wavelength (Å) | 0.5-8.0 (the intensity is maximum at 1.47 Å) | | | |  |
| Beam geometry | Diameter: 14 mm; Sample-to-detector distances: 4.5 m / 12.97 m | | | |  |
| Sample configuration | quartz cell 20 × 50 × 1 mm^3^ | | | |  |
| q-measurement range (Å^-1^) | 0.007 – 0.5 | | | |  |
| Absolute scaling method | Vanadium standard | | | |  |
| Basis for normalization to constant counts | To transmitted intensity by direct beam counter | | | |  |
| Exposure time | 2.0 h | 3.5 h | 3.5 h | 2.0 h |  |
| Sample temperature (°C) | 20 | | | |  |
| **(*c*) Software employed for SAS data reduction, analysis and interpretation** | | | | |  |
| SAS data averaging and subtraction | SAS program for two-detector system | | | |  |
| Calculation of ε from sequence | ProtParam: <https://web.expasy.org/protparam/> | | | |  |
| Calculation of $\bar{\nu}$ values from chemical composition | Peptide Property Calculator: <http://biotools.nubic.northwestern.edu/proteincalc.html> | | | |  |
| Calculation of $\bar{\rho}$ values from chemical composition | SLD calculator web: <https://sld-calculator.appspot.com/> | | | |  |
| Guinier, *P*(*r*) | GNOM from ATSAS | | | |  |
| Atomic structure modelling | MEMPROT 2.2; CRYSON | | | |  |
| Molecular graphics | VMD 1.9.3 | | | |  |
| **(*d*) Structural parameters** | | | | |  |
| Guinier analysis | *Np*SRII/*Np*HtrII  at 0.15 M NaCl | *Np*SRII/*Np*HtrII  at 1.4 M NaCl | *Np*SRII/*Np*HtrII at 2.8 M NaCl | *Np*SRII/*Np*HtrII  at 4 M NaCl |  |
| I(0) (cm^-1^) | 1.1 ± 0.3 | 1.7 ± 0.7 | 2.4 ± 0.5 | 3.2 ± 0.6 |  |
| R_G_ (Å) | 71 ± 28 | 99 ± 37 | 93 ± 19 | 89 ± 17 |  |
| q R_G_ – range | 0.76 – 1.17 | 0.85 – 1.39 | 0.77 – 1.39 | 0.96 – 1.39 |  |
| *P*(*r*) analysis |  |  |  |  |  |
| I(0) (cm^-1^) | 1.13 ± 0.17 | 1.75 ± 0.11 | 2.45 ± 0.15 | 3.53 ± 0.11 |  |
| R_G_ (Å) | 78 ± 18 | 105 ± 5 | 107 ± 5 | 109 ± 2 |  |
| d_max_ (Å) | 350 | 365 | 375 | 390 |  |
| q-range (Å^−1^) | 0.00891-0.260 | 0.00855-0.251 | 0.00830-0.250 | 0.00745- 0.285 |  |
| q_min_ d_max_ / π | 0.993 | 0.993 | 0.991 | 0.925 |  |
| Total quality estimate (GNOM) | 0.411 | 0.555 | 0.5582 | 0.346 |  |
| ***(e)* Atomistic modelling** | | | | |  |
|  | *Np*SRII/*Np*HtrII at 0.15 M NaCl | *Np*SRII/*Np*HtrII  at 1.4 M NaCl | *Np*SRII/*Np*HtrII at 2.8 M NaCl | *Np*SRII/*Np*HtrII  at 4 M NaCl |  |
| Method | CRYSON | OLIGOMER was used for a set of curves calculated for dimers and trimers of dimers using CRYSON | | |  |
| q-range for fitting | 0.00891-0.260 | 0.00855-0.251 | 0.00830-0.250 | 0.00745-0.285 |  |
| Any measures of model precision | Atomic models of dimers and trimers of dimers of the Sensory rhodopsin / transducer complex were generated as described in Materials and Methods.  Pseudo-atomic detergent belt models were generated using MEMPROT program and modified as described in Materials and Methods. | | | |  |
| Background subtraction (cm^-1^); Contrast of the solvation shell (10^‑6^ Å^‑2^); Average atomic radius (Å) | 0.625 × 10^-3^;  0.000; 1.781 | "Tripod"-shaped model:  0.000 × 10^-3^;  0.2056; 1.743 | "Tripod"-shaped model:  0.933 × 10^-3^;  0.4308; 1.701 | "Transmembrane-bound" model:  1.647 × 10^-3^;  "Tripod"-shaped model:  0.707 × 10^-3^;  0.6187; 1.665 |  |
| Volume fraction of trimers of dimers | 0% | "Tripod"-shaped model:  18.3 ± 2.7 % | "Tripod"-shaped model:  28.0 ± 2.2 % | "Transmembrane-bound" model:  23.4 ± 2.2 %;  "Tripod"-shaped model:  36.4 ± 1.9 % |  |
| χ^2^ value; *P* value (from CorMap) | 0.706; 0.0926 | "Tripod"-shaped model:  0.630; 0.2633 | "Tripod"-shaped model:  0.79; 0.0175 | "Transmembrane-bound" model:  5.4; 0.0007  "Tripod"-shaped model: 1.33; 0.0247 |  |
| ***(f)* Data and model deposition IDs** | |  | | |  |
|  | *Np*SRII/*Np*HtrII at 0.15 M NaCl | *Np*SRII/*Np*HtrII  at 1.4 M NaCl | *Np*SRII/*Np*HtrII at 2.8 M NaCl | *Np*SRII/*Np*HtrII  at 4 M NaCl |  |
|  | SASDK37 | SASDK47 | SASDK57 | SASDK67 |  |

**^1^** Parameters were calculated for the dimer of the *Np*SRII/*Np*HtrII complex without detergent belt.

**^2^** Parameters were calculated excluding deuterium exchange.

**Table S3.** Data validation metrics related to SASBDB depositions.

|  | Experimental data range **^1^** | | P(r) fit to data | | Fit validation | |
| --- | --- | --- | --- | --- | --- | --- |
|  | q_min_ d_max_ / π | N_Shannon_ | p_cormap_ | χ² | p_cormap_ | χ² |
| SASDKZ6 | 0.8 (0.673) | 16 | 0.942 | 1.194 | 0 | 1.524 |
|  | 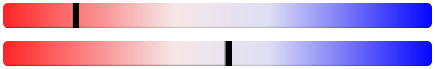 | | 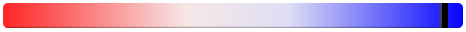 | | 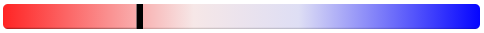 | |
| SASDK27 | 1.0 (0.725) | 43 | 0.946 | 1.040 | 0 | 3.435 |
|  | 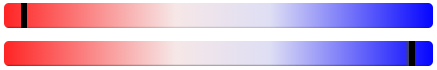 | | 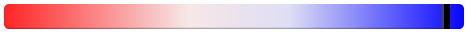 | | 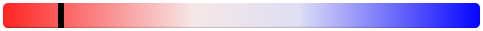 | |
| SASDK37 | 1.2 (0.993) | 28 | 0.182 | 0.389 | 0.093 | 1.588 |
|  | 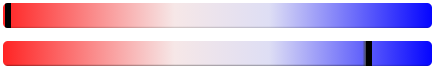 | | 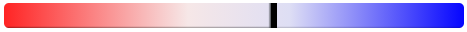 | | 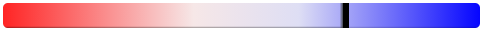 | |
| SASDK47 | 1.0 (0.993) | 27 | 0.138 | 0.526 | 0.263 | 0.631 |
|  | 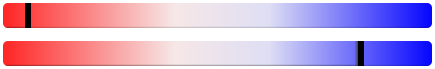 | | 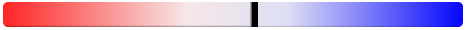 | | 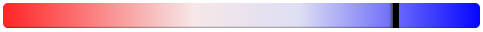 | |
| SASDK57 | 1.0 (0.991) | 30 | 0.070 | 0.500 | 0.018 | 0.793 |
|  | 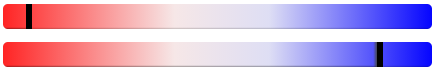 | | 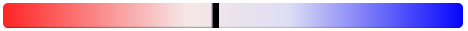 | | 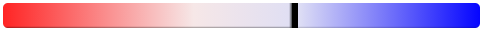 | |
| SASDK67 | 1.3 (0.925) | 35 | 0.001 | 0.897 | 0.025 | 1.330 |
|  | 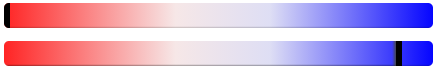 | | 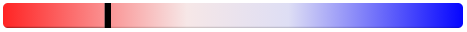 | | 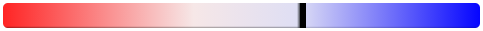 | |

**^1^** The value of the q_min_ d_max_ / π is defined with q_min_ taken for Guinier approximations. Values obtained with q_min_ taken for P(r) and for model fits are shown in brackets (the condition q_min_ d_max_ / π < 1 is right for all of them).
